# Eliciting parents’ decision-making to antibiotic use for upper respiratory tract infections: A discrete choice experiment

**DOI:** 10.7189/jogh.14.04220

**Published:** 2024-12-09

**Authors:** Lixia Duan, Rujiao Lin, Dan Wang, Xi Wang, Xinyi Zhang, Liping Ding, Chenxi Liu

**Affiliations:** 1 Zhejiang Provincial People’s Hospital, Hangzhou, China; 2 School of Medicine and Health Management, Tongji Medical School, Huazhong University of Science and Technology, Wuhan, China; 3 School of Management, Hubei University of Chinese Medicine, Wuhan, China

## Abstract

**Background:**

Addressing antibiotic resistance is important for reducing parents’ self-medication of antibiotics for children’s upper respiratory tract infections (URTIs). However, the decision-making process for parents who irrationally use such antibiotics is still unclear. In this study, we aimed to explore the reasons why parents self-medicate antibiotics for children’s URTIs based on a discrete choice experiment.

**Methods:**

We conducted a systematic review and in-depth interviews to identify the key attributes of choices when parents self-medicate antibiotics for children’s URTIs. We developed and applied a discrete choice experiment in Wuhan and Chongqing, China. We used a mixed logit model to determine the impact of various attributes on parents’ decisions, while we applied latent class logit models to explore different decision-making patterns within populations.

**Results:**

A total of 400 valid responses were returned from parents. It was shown that symptom severity was the most important in parents’ decision-making to self-medicate antibiotics for children’s URTIs, followed by risk of side effects or resistance, duration, total cost, onset time of antibiotic, and antibiotic effectiveness. More severe and longer symptoms, perceived higher effectiveness, and fewer side effects of antibiotics consistently were significantly associated with parents’ more likely to self-medicate with antibiotics for children’s URTIs. There are also different patterns of decision-making of parents, including ‘symptoms-oriented,’ ‘safety-oriented,’ and ‘comprehensive consideration.’ Parents’ gender and educational level were associated with decision-making patterns.

**Conclusions:**

Parents’ self-medication of antibiotics for children’s URTIs was mainly driven by symptoms, followed by perceived antibiotic value. We recommend a multi-faceted intervention strategy to enhance parents’ ability to differentiate mild from severe URTIs, as well as their knowledge of antibiotics.

Antimicrobial resistance (AMR) is considered to be one of the leading global health issues, posing great threats to public health and economic development worldwide. It is estimated that AMR was responsible for 4.95 million deaths in 2019, with 1.27 million of them directly attributed to AMR [1]. Without effective interventions, the projected number of AMR-related deaths is expected to reach 10 million by 2050, leading to economic losses of USD 100 trillion globally [2].

One main contributor to AMR is the irrational and excessive use of antibiotics [3,4], especially for children’s upper respiratory tract infections (URTIs) [5]. Despite most URTIs being caused by viruses, antibiotics were commonly prescribed in the paediatric department for URTIs worldwide, including China, with the rate of antibiotic prescriptions for URTIs exceeding 55% among children aged <5 years [6]. Moreover, self-medication with antibiotics for children’s URTIs by parents was alarmingly prevalent, accounting for approximately 40.5% of antibiotic use in a survey of 3188 parents from three Chinese provinces [7].

Understanding parents’ decision-making of antibiotic self-medication for children’s URTIs is crucial for developing effective strategies to combat antibiotic resistance. Existing studies have mainly focused on identifying factors influencing inappropriate antibiotic use through factor analysis or based on behaviour theories such as the knowledge-attitude-practice model. These studies have identified sociodemographic characteristics (such as age and gender) [8,9], knowledge and attitudes towards antibiotic use [10,11], disease severity [12–14], efficacy of antibiotics in alleviating symptoms [7], as well as external environmental conditions (such as access to antibiotics) [15] as key factors influencing parental decision to use antibiotics when faced with URTIs. Despite implementing interventions based on this evidence, inconsistent effects have been observed.

To further explore the mechanisms behind antibiotic use behaviours, some studies have employed both quantitative [16] and qualitative methods [17] based on behavioural theories. For instance, the application of the health belief model and fuzzy-trace theory have revealed that decisions to use antibiotics for respiratory infections are based on a comprehensive assessment of health beliefs concerning the disease and antibiotics, as well as the individual’s actual circumstances [16,17]. Individuals’ perceptions of the disease and antibiotics influence their assessment of the URTIs’ severity, susceptibility, the perceived effectiveness of antibiotic treatment, and the barriers to antibiotic use. When faced with URTI symptoms such as coughing, individuals weigh the potential benefits and risks of antibiotic treatment before making a decision [16,17].

Qualitative studies using methods such as focus group discussions, semi-structured interviews, and phenomenology have explored the experiences and viewpoints of parents and adults concerning URTIs, medical treatment, and antibiotic use [18–21]. These studies have identified key factors affecting irrational medical treatment and antibiotic use, including individuals’ disease cognition, perceived disease severity, and self-efficacy in managing URTI symptoms. However, these studies were not often supported by robust quantitative data, leaving the key factors involved in parents’ decision-making process to self-medicate antibiotics for children’s URTIs remain unclear.

To address this issue, we conducted a discrete choice experiment (DCE) to comprehensively understand the decision-making processes of parents regarding antibiotic use for their children’s URITs. The DCE, as a quantitative tool, evaluates the preferences and trade-offs of parents by simulating real-world decision-making scenarios [22]. DCE has been widely used in health care and economics and is increasingly applied to explore decision-making processes related to disease management [23,24]. The strength of DCE lies in its ability to systematically identify key attributes influencing parental decisions and quantify the relative importance of these attributes in the decision-making process [25].

Therefore, in this study, we aimed to elicit parents’ decision-making process regarding their choice to self-medicate antibiotics for children’s URTIs in China based on DCE. By integrating qualitative insights from systematic reviews and interviews, we aimed to provide a more comprehensive understanding of the factors that drive parental decisions and contribute to the inappropriate use of antibiotics.

## METHODS

### Study design

We conducted and reported the DCE study following the recommended best practices and guidelines [26]. In the initial phase, we aimed to identify the relevant attributes of parents’ decision-making regarding antibiotic use for URTIs. The subsequent phase involved a quantitative DCE study in which parents of children aged <12 years were presented with various antibiotic alternatives featuring different attribute levels to collect data regarding their decision-making process.

### Identification of attributes and levels

Identifying attributes and levels for the decision-making of parents’ self-medication of antibiotics for children’s URTIs is a key step of the DCE design [27]. Therefore, we first conducted a systematic review to identify the attributes and levels influential in parents’ antibiotic use behaviour for children with URTIs. We identified 14 attributes potentially affecting parents’ decision to use antibiotics for children’s URTIs. These attributes encompassed factors such as disease severity, duration of the illness, specific symptoms, disease awareness, antibiotic effectiveness, onset time of antibiotics, risk of side effects or antibiotic resistance, conditions for antibiotic storage, past antibiotic treatment history, sources of advice for antibiotic use, sources of antibiotics, time required to obtain antibiotics, total cost of obtaining antibiotics, and waiting time for treatment. The details of the systematic review are available elsewhere [28]. Furthermore, we conducted semi-structured interviews between January and April 2022 with 15 purposely sampled parents from a kindergarten in Wuhan, Hubei. These interviews collected parents’ decision-making process regarding antibiotic use for children’s URTIs, aiming to supplement the data from the systematic review. Finally, we encoded the data from systematic review and interviews using thematic analysis, identifying seven attributes with a maximum level of four (**Table 1**, Appendix S1 in the **Online Supplementary Document**).

**Table 1:** Attributes and levels

Attribute	Levels	Definition
Severity of symptoms	Mild, moderate, severe	The severity of symptoms.
Duration of symptoms	1 d, 3 d, 5 d,7 d	The number of days that symptoms have been present, starting from the onset of symptoms
Onset time of antibiotic	2 h, 4 h,8 h	The time it takes effect for antibiotics to take effect
Antibiotic effectiveness	Half cured (50%), basic recovery (90%)	The degree to which symptoms are relieved after taking antibiotics
Risk of side effects or resistance	No risk (0%), low risk (10%), moderate risk (50%), high risk (100%)	The possibility of adverse reactions or antibiotic resistance after taking antibiotics
Time spent obtaining antibiotics	0.5 h, 2 h, 4 h, 6 h	The time required to obtain antibiotics
Total cost	CNY 50, CNY 150, CNY 250, CNY 400	The total cost incurred during the process of obtaining antibiotics, including transportation costs, medication costs, or fees incurred during the medical consultation process.

### Experimental design and construction of choice sets

We developed an unlabelled choice set based on the seven attributes, and each consisted of two antibiotic alternatives plus an opt-out labelled ‘neither’. The opt-out option was applied to reflect real-life scenarios, recognising that not all parents opt for antibiotics when faced with URTIs. To simplify participation and ensure statistical efficiency, we utilised a main effect orthogonal design to generate 24 choice sets using Ngene, version 1.2 (ChoiceMetrics, Sydney, New South Wales, Australia). We divided these choice sets into three blocks, each containing eight choice sets. Additionally, we included a duplicate choice set as a rationality or internal consistency check to ensure respondent’s engagement and seriousness.

The questionnaire consisted of three sections (Appendix S2 in the **Online Supplementary Document**). The first section began with a warm-up choice set to help respondents grasp the DCE survey. The next segment involved a forced selection process with nine choice sets (**Table 2**). The second section explored parents’ knowledge and attitudes towards antibiotic use. This involved assessing their knowledge of antibiotic use and antibiotic resistance (eight items) and exploring attitudes that may impact antibiotic use, such as self-efficacy (five items), social influences (three items), antibiotic use habits (three items), and antibiotic accessibility (three items). The third section gathered the sociodemographic characteristics of parents and their children, including age, gender, education, occupation, medical background, household income level, medical insurance, presence of chronic disease, and the child’s age, gender, and health status.

**Table 2:** Sample choice set presented to the parents

Attributes	Alternative A	Alternative B	Neither
Severity of symptoms	Moderate	Severe	
Duration of symptoms in days	5	1	
Onset time of antibiotic in hours	2	4	
Antibiotic effectiveness (%)	50	90	
Risk of side effects or resistance (%)	High (90)	Low (10)	
Time spent obtaining antibiotics in hours	6	2	
Total cost in CNY	400	150	
Which alternative do you choose for your child?	Yes/no	Yes/no	Neither

We conducted a pilot study in July 2022 with 20 randomly recruited parents from Wuhan, Hubei province, to evaluate the questionnaire’s clarity and acceptability. Based on the feedback received, we made minor modifications to the wording, such as the description of task instructions.

### Discrete choice experiment survey

We used the equation developed by Orme to determine the sample size, considering the number of choice sets and the maximum of attribute levels. Each block in the study consisted of nine choice sets, with two alternatives in each choice set and a maximum of four levels. Therefore, a sample size of 112 was required for each questionnaire version. Considering potential invalid samples and the need to improve statistical efficiency, a sample size of 400 respondents was deemed appropriate for three versions. We included parents aged >20 years with children aged <12 years who had experienced URTI symptoms (such as sore throat, cough, runny nose, and fever). We excluded respondents if they had concomitant lower respiratory tract infections. We used a multi-stage cluster random sampling strategy to select the participants in August 2022, with two to three districts or counties randomly chosen in Wuhan and Chongqing cities based on socio-economic development and geographic location, respectively. For each district or county, we randomly recruited 12–25 samples from regional primary health care institutions. We assigned each respondent to one of the three blocks and conducted a face-to-face questionnaire interview, resulting in 462 samples, with 400 valid samples.

The formal survey was conducted by a team of 10 graduate students from the Social Medicine and Health Services Management program. Before initiating the survey, they underwent a comprehensive one-day training led by the project leader, covering essential topics such as antibiotic use, resistance dynamics, discrete choice experiments, and an overview of the survey questionnaire. A team of two to three researchers managed the survey. Participants completed a self-administered questionnaire preceded by a clear explanation of discrete choice experiment questionnaire instructions (Appendix S3 in the **Online Supplementary Document**). We randomly assigned consenting respondents to one of the three blocks, with an average completion time of 10–15 minutes. Researchers promptly reviewed each submitted questionnaire for accuracy, correcting any errors in collaboration with respondents to ensure strict quality control.

### Statistical analysis

To ensure the reliability and validity of data analysis, we excluded samples with missing values in the sections related to demographics, knowledge, and attitudes towards antibiotic use. We also removed samples that did not meet the consistency check criteria, where all choice sets were uniformly marked as ‘neither option,’ or where all choice sets exclusively selected option A or B.

We performed a descriptive analysis of demographic characteristics, knowledge, and attitudes toward antibiotic use. Continuous variables that conform to a normal distribution are presented as means and standard deviations (SD), while categorical variables are presented as frequencies and percentages.

We used the mixed logit model (MLM) to analyse the DCE survey data. The MLM is particularly adept at handling the independence of irrelevant alternatives, suggesting that the presence or absence of an option can influence the relative attractiveness of the remaining choices. The MLM approach allows flexibility in recognising that participants’ preferences may differ. This was achieved by allowing the parameters to vary randomly across individuals, capturing individual heterogeneity through the distribution of the model parameters. The dependent variable, representing the choice to make a claim, was binary, with ‘1’ for ‘yes’ and ‘0’ for ‘no’. All variables were categorical, and we dummy-coded them according to the definitions described in **Table 1**. In the dummy variable encoding, one level of each attribute is systematically excluded to prevent multicollinearity. We employed a binary coding scheme for each non-excluded attribute level. It is coded as ‘1’ when that level is present in a given choice scenario and ‘0’ when another level of the same attribute is presented. All attributes and the constant were specified to have random coefficients that were normally distributed. The coefficients’ means in the MLM represent the estimated average preference weights. The magnitude of the coefficients indicates the strength of preference for the levels of an attribute. A positive coefficient suggests a preference for that level compared to the reference level, while a negative coefficient indicates a less favourable preference. The SD reflects the degree of heterogeneity among individuals; if the differences in SD are statistically significant, it indicates heterogeneity in individual preferences, which can be further analysed by including interaction terms to examine the impact of different sociodemographic characteristics and perceptions on preferences [29,30]. We calculated the relative importance of each attribute using model coefficients. This involved taking the absolute value of the estimated means for each attribute parameter, multiplying it by the difference between the highest and lowest attribute levels, and comparing the resulting value (maximum effect) to the total for each attribute to determine its relative importance.

We used the latent class logit model (LCLM) to analyse preference heterogeneity at the group level. The LCLM analysis involves categorising participants into several groups to achieve homogeneity within each group and heterogeneity between different groups [29,30]. We determined the number of categories using various goodness-of-fit indices, with lower values of Akaike information criterion, Bayesian information criterion, and consistent Akaike information criterion indicating better model fit and higher values of log-likelihood value representing better level of fitness. For interpreting LCLM results, the significance of the attribute coefficients is similar to that in MLM. It is important to note that attribute coefficients are comparable within a single class but not directly comparable across different classes [29,30]. We further subjected the classes derived from the LCLM to regression analysis with demographic characteristics to explore the impact of different demographic traits on each latent class. Data analysis was conducted using ‘mixlogit’ and ‘lclogit2’ commands in Stata, version 14.0 (StataCorp LLC, College Station, Texas, USA).

### Ethical approval

The Research Ethics Committee of Tongji Medical College, Huazhong University of Science and Technology, approved this study (approval number IORG0003571).

## RESULTS

### Characteristics of the respondents

A total of 400 parents participated in this study. The average age of parents was 35.63 years, with a predominance of female participants. A significant majority (82.00%) boasted a high school education or higher, reflecting a well-educated sample group (**Table 3**). Occupational diversity was evident, yet a notable 58.25% reported an annual household income below CNY 120 000, offering a glimpse into the economic context of the study’s population. Regarding medical insurance, more than half of the respondents (53.25%) were enrolled in urban employee medical insurance, a critical factor influencing health care access and decision-making. Most respondents (79.25%) and their families had no medical background. Health concerns were present, with 20.25% of families coping with chronic diseases. The children’s sample, evenly split by gender, had a mean age of six years, an age group critical for early health interventions and education. The majority of children, 73.50%, were in good health, indicating a generally healthy study population, but it also highlighted the need to maintain this status through informed health care decisions.

**Table 3:** Demographic characteristics of parents (n = 400) in the discrete choice experiment*

Respondent characteristics	n (%)
Age in years, x̄ (SD)	35.63 (6.35)
Gender	
*Female*	324 (81.00)
*Male*	76 (19.00)
Education	
*Junior high school and below*	72 (18.00)
*Senior high school and vocational education*	77 (19.25)
*Associate degree education*	91 (22.75)
*Undergraduate education and above*	160 (40.00)
Occupation	
*Farmers*	30 (7.50)
*Workers*	25 (6.25)
*Students*	4 (1.00)
*Medical professionals*	29 (7.25)
*Teachers*	21 (5.25)
*Staff in government agencies, enterprises and institutions*	61 (15.25)
*Self-employed*	72 (18.00)
*Retired*	4 (1.00)
*Unemployed*	51 (12.75)
*Other*	103 (25.75)
Annual income in CNY	
*<40 000*	65 (16.25)
*40 000–80 000*	82 (20.50)
*80 000–120 000*	86 (21.50)
*>120 000*	167 (41.75)
Medical insurance	
*Urban employee basic medical insurance*	213 (53.25)
*Rural and urban resident basic medical insurance*	168 (42.00)
*Other*	19 (4.75)
Medical background	
*Yes*	81 (20.25)
*No*	319 (79.25)
Chronic disease	
*Yes*	111 (27.75)
*No*	289 (72.25)
Children’s gender	
*Female*	196 (49.00)
*Male*	204 (51.00)
Children’s age in years, x̄ (SD)	6.43 (3.54)
*<3*	65 (16.25)
*3–6*	85 (21.25)
*6–9*	132 (33.00)
*>9*	118 (29.50)
Children’s health status	
*Healthy*	294 (73.50)
*Good*	88 (22.00)
*Moderate*	11 (2.75)
*Poor*	6 (1.50)
*Very poor*	1 (0.25)

SD – standard deviation, x̄ – mean

*Presented as n (%) unless specified otherwise.

### Knowledge and attitudes towards antibiotic use among the parents

Our findings indicate a concerning lack of comprehensive knowledge among parents regarding antibiotic use and resistance, with an overall accuracy rate of merely 49.75% (**Table 4**). While a significant majority (86.25%) demonstrated a good understanding of bacterial resistance, there was a notable gap in knowledge concerning the distinction between antibiotics and anti-inflammatory drugs and the ineffectiveness of antibiotics for viral infections, with accuracy rates between 36.25–38.50%. Alarmingly, only 8.00% of parents correctly recognised that the human body does not develop resistance to antibiotics, a fundamental misconception with significant public health implications.

**Table 4:** Parents’ knowledge of antibiotic use*

Items	n (%)
Antibiotics are effective in treating most colds. (false)	165 (41.25)
Antibiotics are synonymous with inflammatory drugs. (false)	154 (38.50)
Antibiotics are effective in treating viral colds. (false)	145 (36.25)
Antibiotics are effective in treating bacterial colds. (false)	293 (73.25)
The human body develops resistance to antibiotics. (false)	32 (8.00)
Bacteria develop resistance to antibiotics. (true)	266 (66.50)
Overuse of antibiotics can lead to antibiotic resistance. (true)	345 (86.25)
As long as the usage is short, there will be no antibiotic resistance. (false)	190 (47.50)
Total score, x̄ (SD)	3.98 (1.82)

SD – standard deviation, x̄ – mean

*Presented as n (%) unless specified otherwise.

Parents showed a high level of self-efficacy (mean (x̄) = 2.89; 95% confidence interval (CI) = 2.11, 3.67) in their ability to use antibiotics, indicating that they believed they had sufficient knowledge of rational antibiotic use, were capable of treating minor symptoms with antibiotics independently, and had confidence in self-diagnosis and treatment for colds. However, parents scored slightly lower in terms of knowing when to use antibiotics and making judgments about antibiotic use without consulting a doctor. This suggests a potential overconfidence that could lead to improper antibiotic use without sufficient medical advice (**Table 5**).

**Table 5:** Parents’ attitudes of antibiotic use

	x̄ (SD)
**Self-efficacy**	2.89 (0.78)
I think I know enough about the rational use of antibiotics.	2.89 (0.92)
I think I’m capable of taking antibiotics to deal with the milder symptoms on my own.	2.91 (1.04)
I usually have confidence in self-diagnosis and the treatment of colds on my own.	2.98 (1.00)
I usually know when antibiotics are needed.	2.88 (1.02)
I usually know if I need antibiotics for a cold before I go to doctor.	2.78 (1.01)
Social influence	2.52 (0.74)
The doctor prescribed antibiotics for my cold in the past.	2.71 (0.91)
Friends and family recommended that I use antibiotics to treat my cold.	2.29 (0.96)
The pharmacy recommended that I purchase antibiotics to treat my cold.	2.71 (1.02)
The antibiotic use for treating or preventing a cold is a common practice.	2.38 (1.03)
Availability of antibiotics	2.64 (1.03)
I can easily purchase antibiotics from a pharmacy without prescription.	2.92 (1.22)
I have never been asked to provide a prescription when buying antibiotics.	2.49 (1.40)
I can easily obtain antibiotics from my family, friends or from home medicine cabinets.	2.51 (1.23)
Antibiotic use habit in the past year	2.10 (0.81)
Have you ever used antibiotics to prevent disease deterioration when the illness was not yet severe?	1.87 (0.95)
Have you ever taken antibiotics before seeking medical attention?	1.88 (0.95)
Have you ever purchased and self-administered antibiotics from a pharmacy without seeking medical care?	1.99 (0.97)
Do you keep antibiotics readily available in your household?	2.64 (1.23)

SD – standard deviation, x̄ – mean

The moderate level of social influence (x̄ = 2.52; 95% CI = 1.78, 3.26) indicates that health care professionals are crucial in guiding parental decisions. They were greatly influenced by doctors and pharmacies, who prescribed antibiotics or recommended them at pharmacies.

The ease of access to antibiotics (x̄ = 2.64; 95% CI = 1.61, 3.67), often without a prescription, raises concerns about the over-the-counter availability of these drugs. They could easily purchase antibiotics from pharmacies, family, friends, or home medical supplies. When purchasing antibiotics at a pharmacy, they were rarely required to present a doctor’s prescription.

As for antibiotic use habits in the past year, the frequent storage of antibiotics in homes, despite infrequent use as a preventive measure, may encourage inappropriate self-medication and contribute to the problem of unused medications.

### Parents’ antibiotic decision-making using mixed logit models

The study utilising mixed logit models sheds light on the factors influencing parents’ decisions to administer antibiotics for their children’s URTIs. The results underscore the critical role of symptom severity, with parents significantly more likely to opt for antibiotics when symptoms are severe (Beta coefficient (*β*) = 2.46; *P* < 0.05), as opposed to mild symptoms. This inclination is also evident for moderate symptoms (*β* = 1.11; *P* < 0.05), highlighting a direct correlation between the perceived severity of illness and the likelihood of antibiotic use (**Table 6**).

**Table 6:** Parental antibiotic preference using mixed logit models

Attributes	*β* (SE)	*P*-value	SD (SE)	*P*-value
Symptoms				
*Mild*	Ref		Ref	
*Moderate*	1.11 (0.13)	<0.0001	–0.41 (0.19)	0.036
*Severe*	2.46 (0.19)	<0.0001	1.96 (0.17)	<0.0001
Duration in days				
*1*	Ref		Ref	
*3*	0.99 (0.18)	<0.0001	–0.49 (0.22)	0.026
*5*	0.99 (0.18)	<0.0001	0.24 (0.21)	0.254
*7*	1.02 (0.18)	<0.0001	0.75 (0.19)	<0.0001
Antibiotic effectiveness in %				
*50*	Ref		Ref	
*90*	0.35 (0.14)	0.011	0.61 (0.12)	<0.0001
Onset time of antibiotic in hours				
*2*	Ref		Ref	
*4*	0.13 (0.14)	0.347	0.04 (0.17)	0.806
*8*	0.36 (0.14)	0.013	–0.01 (0.21)	0.954
Risk of side effects or resistance				
*No risk*	Ref		Ref	
*Low*	0.03 (0.14)	0.818	1.00 (0.17)	<0.0001
*Moderate*	–0.44 (0.16)	0.006	–0.07 (0.24)	0.766
*High*	–1.13 (0.21)	<0.0001	1.17 (0.20)	<0.0001
Time spent obtaining antibiotics in hours				
*0.5*	Ref		Ref	
*2*	0.32 (0.21)	0.129	0.42 (0.24)	0.084
*4*	0.32 (0.17)	0.064	–0.25 (0.40)	0.530
*6*	0.24 (0.19)	0.216	–0.89 (0.19)	<0.0001
Total cost in CNY				
*50*	Ref		Ref	
*150*	0.45 (0.16)	0.005	–0.56 (0.23)	0.016
*250*	0.44 (0.15)	0.003	–0.08 (0.20)	0.682
*400*	0.48 (0.18)	0.010	–0.01 (0.20)	0.977
ASC	–0.05 (0.62)	0.930	2.49 (0.21)	<0.0001
Sample (n)	400			
Observation (n)	9600			
Log-likelihood	–2470.2682			

ASC – alternative specific constant, ref – reference, SD – standard deviation, SE – standard error, *β* – beta coefficient

The duration of symptoms also significantly influences parental decisions, with a strong preference for antibiotic use emerging when symptoms persist for seven days (*β* = 1.02; *P* < 0.05). This preference is slightly less pronounced but still significant for durations of three days (*β* = 0.99; *P* < 0.05) and five days (*β* = 0.99; *P* < 0.05).

Parents preferred antibiotics with better perceived effectiveness (*β* = 0.35; *P* < 0.05), indicating a desire for tangible relief treatments. Additionally, the onset time of antibiotic use within eight hours (*β* = 0.36; *P* < 0.05) was favoured over a two-hour window, suggesting their different perspectives on the timing of treatment rather than simply emphasising immediate treatment. Interestingly, parents preferred antibiotics with a lower risk of side effects or resistance (*P* < 0.05), reflecting a cautious approach to potential health risks.

Economic factors play a significant role in parental decision-making, with a notable preference for higher-cost antibiotics. Parents showed a significant inclination towards antibiotics priced at CNY 400 (*β* = 0.48; *P* < 0.05) and CNY 150 (*β* = 0.45; *P* < 0.05), compared to a lower-cost option of CNY 50. This preference for higher-priced antibiotics may stem from a belief that cost indicates quality or effectiveness, a perception that warrants further investigation and education. No significant preference was identified for the time spent obtaining antibiotics.

Heterogeneity in preference for these attributes was observed based on the standard deviation, indicating that factors such as gender, educational background, annual family income, medical background, knowledge of antibiotics, self-efficacy level, and social influence level significantly shape parental decisions. For example, compared to mild symptoms, mothers (*β* = 0.71; *P* = 0.036), parents with a medical background (*β* = 0.82; *P* = 0.019), and parents with education levels above a bachelor’s degree (*β* = 0.64; *P* = 0.033) exhibited a greater propensity to administer antibiotics to their children during severe symptoms. Parents with higher self-efficacy were less inclined to use antibiotics for their children when symptoms persisted for five days (*β* = –0.51; *P* = 0.009) or seven days (*β* = –0.50; *P* = 0.018), in contrast to a duration of one day. This diversity underscores the need for tailored educational interventions and public health messaging that consider the multifaceted nature of decision-making (Appendix S4 in the **Online Supplementary Document**).

**Figure 1:**
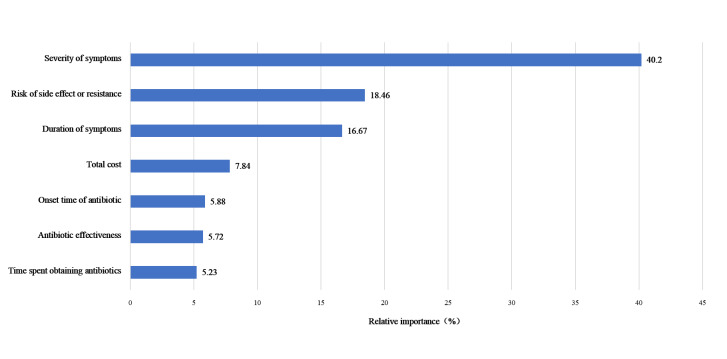
The relative importance.

The relative importance estimates from the mixed logit model indicated that symptom severity was the most important attribute in parents’ decision to use antibiotics, followed by risk of side effects or resistance, duration, total cost, onset time of antibiotic, antibiotic effectiveness, and time spent obtaining antibiotics (**Figure 1**). These insights are crucial for health care providers and policymakers in developing targeted strategies to promote the rational use of antibiotics.

### Parents’ antibiotic preferences using latent logit models

The latent logit model with three latent classes was determined as the optimal model based on the best goodness-of-fit indices (Bayesian information criterion = 5197.9134; Akaike information criterion = 5197.9134; consistent Akaike information criterion = 5253.9134). This model provides a nuanced understanding of parental preferences and decision-making processes regarding antibiotic use for their children.

### Class one: symptom-oriented parents

This class, comprising 105 parents, placed the utmost importance on the severity of symptoms as the decisive factor for antibiotic use, with moderate (*β* = 1.650; *P* < 0.001) and severe (*β* = 3.458; *P* < 0.001) symptoms receiving significant preference (**Table 7**). This class did not exhibit significant preferences for other attributes, indicating a focused prioritisation on symptom severity in decision-making, and was therefore labelled as ‘symptom-oriented.’

**Table 7:** Parental antibiotic preference using latent logit models

Attributes and levels	Class 1, *β* (SD)	Class 2, *β* (SD)	Class 3, *β* (SD)
Symptoms			
*Moderate*	1.650 (0316)*	0.222 (0.124)	1.654 (0.335)*
*Severe*	3.458 (0.388)*	0.192 (0.149)	3.693 (0.331)*
Duration in days			
*3*	0.309 (0.580)	0.382 (0.191)*	1.086 (0.415)*
*5*	0.001 (0.654)	0.249 (0.201)	1.621 (0.408)*
*7*	0.154 (0.644)	0.348 (0.201)	1.367 (0.401)*
Onset time of antibiotic in hours			
*4*	–0.701 (0.486)	–0.017 (0.131)	0.351 (0.270)
*8*	–0.500 (0.642)	0.167 (0.152)	0.152 (0.329)
Antibiotic effectiveness in %			
*90*	–0.389 (0.566)	0.054 (0.154)	0.486 (0.269)
Risk of side effects or resistance			
*Low*	–0.628 (0.412)	–0.155 (0.148)	0.265 (0.326)
*Moderate*	–0.759 (0.480)	–0.612 (0.170)*	–0.044 (0.339)
*High*	–0.709 (0.552)	–1.301 (0.206)*	–0.220 (0.371)
Time spent obtaining antibiotics in hours			
*2*	–1.481 (0.932)	–0.086 (0.221)	0.500 (0.370)
*4*	–0.999 (0.632)	–0.012 (0.174)	0.090 (0.374)
*6*	–1.257 (0.775)	0.091 (0.200)	0.422 (0.394)
Total cost in CNY			
*150*	–0.211 (0.566)	0.103 (0.170)	0.437 (0.295)
*250*	–0.367 (0.511)	0.002 (0.157)	0.558 (0.283)*
*40*	–1.039 (0.822)	0.123 (0.209)	0.308 (0.366)
ASC	3.041 (2.325)	2.315 (0.725)*	–4.710 (1.224)
Constant	0.033 (0.251)	0.648 (0.185)	NA
Class probability	0.485	0.264	0.252
Sample(n)	400		
Observation(n)	9600		
Log-likelihood	–2431.1957		
AIC	4974.3914		
BIC	5197.9134		
CAIC	5253.9134		

AIC – Akaike information criterion, ASC – alternative-specific constant, BIC – Bayesian information criterion, CAIC – consistent Akaike information criterion, NA – not available, SD – standard deviation, *β* – beta coefficient

*P-value <0.05.

### Class two: safety-oriented parents

With 194 parents, this class was primarily concerned with the duration of symptoms and the associated risks of side effects or antibiotic resistance. The preference for a three-day symptom duration was notable (*β* = 0.382; *P* = 0.045), alongside a marked aversion to moderate (*β* = –0.612; *P* < 0.001) and high (*β* = –1.301; *P* < 0.001) risks of side effects or resistance. This class’s label reflects a cautious approach to antibiotic use, emphasising safety and minimising potential harm.

### Class three: comprehensive consideration parents

The smallest class, with 101 parents, demonstrated a multifaceted approach to decision-making, significantly favouring symptom severity and duration and the total cost of antibiotics. Notable preferences were observed for both moderate (*β* = 1.654; *P* < 0.001) and severe (*β* = 3.693; *P* < 0.001) symptoms, symptom durations of three (*β* = 1.086; *P* = 0.009), five (*β* = 1.621; *P* < 0.001), and seven days (*β* = 1.367, *P* = 0.001), and a cost of CNY 250 for antibiotic acquisition (*β* = 0.558; *P* = 0.049). This class’s label indicates a more holistic consideration of various antibiotic use factors.

Demographic associations with class membership were also observed. Mothers were found to be more likely to fall into the ‘safety-oriented’ class, potentially reflecting a heightened concern for the safety and well-being of their children. Additionally, parents with higher levels of education were more inclined to be part of the ‘comprehensive consideration’ class, suggesting that greater educational attainment may be associated with a more nuanced and multifaceted evaluation of antibiotic use.

## DISCUSSION

### Main findings

To our knowledge, this is the first DCE to investigate parental decision-making for antibiotic self-medication in the context of children with URTIs. By analysing trade-off data collected from a sample of 400 parents who faced the decision of whether to use antibiotics for their children’s URTIs, we found that parents were more inclined to choose antibiotics when their children experienced more severe and prolonged symptoms when the onset time of antibiotics was longer, the effectiveness of antibiotic was better, the risk of side effects or resistance was lower, and the total cost was higher. In addition, the heterogeneity in parental antibiotic preferences was observed in parents with different characteristics.

We found that the severity of symptoms was the main contributor to parents’ self-medication of antibiotics for children’s URTIs. When parents perceived symptoms of their children to be severe, their concerns for their child’s health intensified, leading to immediate relief for their child's symptoms [7,12]. Antibiotics were generally regarded as a panacea for alleviating symptoms associated with severe URTIs [7,12]. On the other hand, existing qualitative studies have shown that URTIs in children were often perceived as serious illness due to their susceptibility and uncertainty [31,32]. Therefore, improving parents’ ability to differentiate between mild and severe URTIs and promote appropriate management of URTIs and rational antibiotic use is essential.

The risk of side effects or resistance significantly influenced parents' decision-making to use antibiotics for their children, as shown by studies conducted in various countries [33,34]. For example, a study conducted with 222 adults in Colombia revealed that side effects and the potential for antibiotic resistance were among the most crucial factors in over-the-counter drug purchases [33]. It was estimated that if patients were informed about the risks of antibiotic use and the limited effectiveness of antibiotics for URTIs, the prevalence of self-medication with antibiotics could decrease from 47.3% to 26.5% of the population [33]. Another study in the UK involving 2579 participants assessed treatment preferences for URTIs and found that the public was disinclined to use antibiotics if it increased the risk of antibiotic resistance for family members or future generations [34]. Consequently, enhancing parents’ awareness of the side effects and resistance risks associated with antibiotics is critical to promote rational antibiotic use.

The duration of symptoms was identified as a key factor influencing parents’ decision-making process, with a preference for antibiotic usage observed when symptoms lasted longer. The results were consistent with other studies exploring displayed preferences [35]. Ebell et al. systematic review and empirical comparative study demonstrated that individuals’ expectations for complete resolution of cough symptoms within five to seven days often deviated from the actual course of illness. Consequently, individuals sought medical attention and requested antibiotics when experiencing cough symptoms for five to seven days. However, coughing typically diminished around the seventh day and disappeared within three to four days without antibiotic treatment. When individuals took antibiotics within a five to seven days timeframe, they may attribute the natural progression of the disease to the impact of antibiotics, reinforcing the expectations for antibiotics [14]. Therefore, guiding the actual duration of symptoms associated with URTIs and appropriate treatment measures could assist parents in using antibiotics more reasonably. Interestingly, this result was in contrast with a study conducted in Colombia, where respondents were more inclined to seek medical attention and take antibiotics when symptoms endured for shorter durations [33]. This discrepancy may be attributed to the study’s focus on adults, most of whom were employed and self-administered antibiotics to expedite their return to work and normal routine in Colombia [36].

The impact of the total cost on parents’ decision to use antibiotics was reflected in parents’ willingness to spend more money to access antibiotics. However, Johanna’s study indicated that participants preferred to spend less money on doctors' advice and antibiotic treatment [33]. This disparity aligned with the cultural context of China, where cost concerns were lower due to the prominent role of children within the family [30]. Moreover, a survey in Anhui demonstrated that parents’ concern for their children’s well-being motivated them to seek medical attention and use antibiotics for their children’s URTIs, regardless of the cost [37].

The effectiveness and onset time of antibiotics played a crucial role in parents’ decisions about antibiotic usage. This was consistent with previous DCE, which indicated that the public tends to choose antibiotics that can shorten the duration of the illness and alleviate symptoms more effectively [33,34]. However, a common misconception persisted among the public regarding the effectiveness of antibiotics in treating URTIs [14,38–40].

The time spent for medical treatment did not significantly impact parents’ choice to use antibiotics, which corroborated Johanna’s DCE conducted in Colombia [33]. However, several cross-sectional studies have recognised time spent as an influential factor in self-medication behaviour [41,42]. This outcome could be attributed to two reasons. First, parents prioritise immediate relief of their children’s symptoms over the time needed for medical treatment, making them less sensitive to differences in this attribute. Second, given the numerous attributes included in this study, parents might have overlooked the attribute of time spent.

Furthermore, the results of this study showed that parents with different characteristics demonstrated varying preferences when deciding on antibiotic usage. For example, mothers, parents with medical backgrounds, and those with undergraduate or higher education levels displayed a greater inclination to use antibiotics when their children’s symptoms were more severe. Parents who strongly believed in their self-efficacy in using antibiotics were more willing to use them promptly. Additionally, latent class analysis confirmed the heterogeneity in parents' preferences. Existing cross-sectional studies using latent class analysis also highlighted distinct behavioural patterns of antibiotic use in the population, with the different predictive factors influencing these patterns [43–45]. Therefore, it is crucial to tailor them to a specific target population and address the concerns of each group.

There are several limitations in this study. First, including seven attributes in the DCE might have posed a cognitive burden for some participants. Second, while the relative importance provided valuable insights, we recognised that our analysis was based primarily on the beta coefficients derived from the MLM without conducting significance testing on these coefficients, which may affect the robustness of the relative importance of each attribute. Future research is encouraged to incorporate significance testing to validate these attributes’ influence and provide a more nuanced understanding of the decision-making dynamics. Furthermore, despite efforts to quantitatively present real-life scenarios during the experimental design and implementation, future research is warranted to explore and validate the compatibility of these preferences with actual behaviours. Moreover, the sample population of this study was restricted to Chongqing and Wuhan, and attempts to generalise the results to other conditions should be cautious.

### Implications

We provided a comprehensive understanding of parents’ multifaceted decision-making regarding antibiotic use for their children’s URTIs. The findings highlight the significant roles of perceived severity of symptoms, antibiotic effectiveness, and perceived risks of side effects or antibiotic resistance in shaping parental choices. These insights underscore the need for targeted interventions that curb improper antibiotic use and empower parents with the knowledge and tools to manage URTIs effectively.

Educational interventions should focus on helping parents distinguish between the severity of their child’s symptoms and understand the natural progression of URTIs. Developing user-friendly symptom assessment tools, such as apps or community workshops, can aid parents in making informed decisions. Providing resources to differentiate between viral and bacterial infections, assessing disease severity, monitoring progression, understanding conditions and signals that pose high risks, choosing suitable treatment options, and adopting preventive measures against future URTIs can foster the development of appropriate behaviours for managing URTIs.

Moving from awareness to action, our findings can drive policy influence and decision-making by shaping antibiotic stewardship programs. These policies should incorporate the aforementioned tools and education on symptom management rather than solely emphasising the risks of antibiotic resistance and strict measures [46,47]. Additionally, regulatory measures should be tightened on over-the-counter antibiotic sales and ensure clear warnings about their limited efficacy against viral infections.

To dispel misconceptions about the effectiveness of antibiotics in treating URTIs, targeted communication strategies are recommended, including media campaigns that debunk common myths and doctor-patient communication training. These workshops would enhance health care providers’ ability to discuss antibiotic use and resistance, fostering a more informed dialogue. In the cultural context of China, strategies should highlight the long-term benefits of prioritising children’s health over the uncertain short-term effects of misusing antibiotics. Campaigns like ‘Investing in Your Child’s Future Health’ could resonate with the values and concerns of parents.

Acknowledging the diversity in parental preferences, interventions should be tailored to address the specific concerns of different demographic groups. For example, tailored levels of knowledge and guidance on antibiotic use should be offered, considering the diverse acceptance and comprehension capacities of parents with varying educational backgrounds. Online modules can provide in-depth information for parents with higher education levels, while pictorial guides can cater to those with lower literacy rates. Targeted engagement with mothers, acknowledging their increased vigilance regarding antibiotic resistance, can position them as the decision-makers for antibiotic use within the household. Artificial intelligence-driven tools can further personalise appropriate antibiotic use and resistance risk information.

## CONCLUSIONS

Parents’ decision-making process to use antibiotics for children’s URTIs was significantly influenced by symptom severity, duration of symptoms, antibiotic effectiveness, onset time of antibiotic, risk of side effects or antibiotic resistance, and total cost, while preference heterogeneity was observed in parents with different characteristics. A collaborative intervention strategy that engages health care providers, educators, policymakers, and community leaders is recommended to enhance parents’ knowledge of antibiotics, increase self-efficacy in disease management, and guide the appropriate coping strategies for URTIs. Differentiated interventions considering demographic variations will further support effective antibiotic stewardship.

## Additional material


Online Supplementary Document
