# Supplementary material for: Eliciting parents’ decision-making to antibiotic use for upper respiratory tract infections: A discrete choice experiment
Source: J Glob Health. 2024 Dec 9;14:04220. doi: 10.7189/jogh.14.04220 (PMC11626686; doi:10.7189/jogh.14.04220)
Supplement: Online Supplementary Document [file jogh-14-04220-s001.pdf]

Appendix S1 Identifying Attributes and Levels in a Discrete Choice Experiment (DCE) on Parental Decisions Regarding Antibiotic Use for URTIs in Children

This section outlines the process of identifying the attributes and levels used in a Discrete Choice Experiment (DCE) designed to explore the factors that influence parents' decisions about administering antibiotics for upper respiratory tract infections (URTIs) in their children. The identification process involved a systematic review and in-depth interviews to gain comprehensive insights into antibiotic misuse for URTIs, and to understand the underlying reasons. A thematic analysis of the qualitative data from the interviews revealed the behavioral mechanisms and key attributes related to antibiotic use in URTIs.

Methods

Systematic review

A thorough literature search was conducted across PubMed, Cochrane Library, Embase, and Web of Science using a set of English and Chinese keywords. The search aimed to retrieve studies published before October 28, 2021, that focused on the general population's behavior and knowledge regarding antibiotic use for URTIs. The detailed search strategy is provided in Table S3-1.

[Table S1-1]

Two researchers independently screened the titles and abstracts for relevance to antibiotic use in the general population. Studies were selected for full-text review based on predefined criteria, including the involvement of the general public, adults, the elderly, caregivers, and outpatients, specifically targeting URTIs (diagnosis of upper respiratory tract infection or presence of symptoms like cough, sore throat, rhinorrhea, nasal congestion, headache, and fever). Studies involving lower respiratory infections were excluded.

During data extraction, the researchers meticulously gathered information on study characteristics, methodology, results, and critical appraisal notes. The quality of the literature was assessed using appropriate tools for quantitative studies (JBI quality assessment tool), qualitative studies (Critical Appraisal Skill Programme tool), and mixed-methods studies (Mixed Methods Appraisal Tool). Disagreements were resolved by a third party. Only medium and high-quality studies were included in the final analysis, ensuring a robust evidence base.

In-Depth Interview

A purposeful sampling method was employed to select 15 diverse parents from Wuhan-area kindergartens, considering variables such as gender, age, education, economic status, and

URTI treatment approaches (home treatment, medical consultation, antibiotics). The inclusion criteria were: (1) Over 18 years old with good communication skills and willing to participate; (2) Children under 6 years old; (3) URTI symptoms experienced within the past year; (4) Exclusion of individuals with lower respiratory tract infection symptoms like chronic bronchitis, asthma, or pneumonia.

The interviews were conducted by two investigators, one leading the dialogue and the other documenting. Both interviewers, who had relevant research backgrounds, received professional training and conducted mock interviews to ensure the accuracy of the data. The semi-structured interviews began with open-ended questions, allowing parents to elaborate on their URTI management approaches, treatment decisions, and perceptions of antibiotic efficacy. Interviews were audio-recorded with participant consent. The process continued until information saturation was achieved after 13 interviews, with two additional interviews conducted for confirmation, ultimately including 15 interviewees.

Thematic Analysis

Thematic analysis was conducted on the data collected from the systematic review and in-depth interviews. Following Braun and Clarke's framework[1], the data was meticulously coded through stages of familiarization, initial coding, theme identification, and thorough review to ensure saturation and thematic coherence. Given the variability in quantitative study measurements, a meta-analysis was not feasible; instead, a narrative synthesis was conducted to align the findings with behavioral stages, informing the attribute and level selection for the DCE. MAXQDA software was used to support the analysis, facilitating a deep understanding of key themes and narratives that shaped the experimental design's attributes and levels.

Results of Systematic Review

The systematic review retrieved 8,544 articles, of which 114 were selected based on inclusion criteria. After a rigorous quality assessment, 28 low-quality articles were excluded, leaving 86 high-quality references, including 48 quantitative, 30 qualitative, and 8 mixed-methods studies. Through narrative synthesis, the determinants of inappropriate antibiotic use were categorized into three groups: disease-related, antibiotic-related, and demographic factors. This comprehensive analysis provided a nuanced understanding of the multifaceted influences on antibiotic use behavior, as detailed in Tables S1-2, S1-3, S1-4, and S1-5.

[Table S1-2& S1-3 & S1-4 &S1-5]

Attribute and Level Development

Following Helter and Boehler's framework[2], our study employed a four-step process: initial

data collection, data streamlining, elimination of irrelevant attributes, and definition of the remaining attributes. We began by compiling a comprehensive raw database from our literature review. Thematic analysis was then used to identify the critical behaviors and factors associated with antibiotic use in individuals with URTIs, resulting in a curated list of attributes and levels.

In refining our attribute list, we adhered to Helter's criteria for inclusion, ensuring the attributes were relevant to individual decision-making, distinct from the DCE's structure to avoid deterministic outcomes, and not so dominant as to eliminate randomness in decision-making. Attributes inherently tied to individual personality traits were excluded[2]. This rigorous selection process resulted in 14 preliminary attributes, as outlined in Table S1-6.

[Table S1-6]

Results of Qualitative Interviews

Thematic analysis identified 7 main themes and 16 sub-themes (Table S1-7), including the impact of the epidemic and the decision-making process. The decision-making process encompasses six stages: need identification, information search, treatment plan evaluation, acquisition of antibiotics, antibiotic use, and post-use evaluation.

[Table S1-7]

Need Identification

The public's initial response to URTI symptoms involves recognizing, attributing, and identifying the illness, which guides subsequent treatment decisions. Interviewees demonstrated a strong ability to identify URTIs, recognizing symptoms like nasal congestion, rhinorrhea, sore throat, and cough, as well as non-disease indicators like loss of appetite and a sensation of obstruction in the throat. Attributions for URTIs varied, with some linking them to environmental factors (such as weather fluctuations, temperature extremes, chilly winds, and exposure to rain) and others to infectious sources. It's not uncommon for both environmental and infectious factors to coexist. Most interviewees did not consider URTI symptoms to be severe, with a few even viewing occasional URTIs as beneficial for immune system strengthening.

Interviewees evaluate the severity of an infection by drawing on their individual experiences and adjusting their treatment strategies to suit. They generally assess the severity through three distinct perspectives:

Specific Symptoms: The appearance of atypical or alarming symptoms—such as persistent headaches or fevers—elevates the level of concern for the infection's severity.

Symptom Progression: A decline in the condition of existing symptoms (for instance, a sore

throat becoming more painful, or a mild fever intensifying), the evolution from a singular symptom to a cluster, or the failure of attempted remedies, all indicate a more grave medical situation.

Symptom Impact: Symptoms that persist beyond the expected timeframe or significantly interfere with one's daily life and professional obligations are considered serious.

There is a lack of uniformity among the interviewees regarding the benchmarks for disease severity, and what is perceived as serious can differ markedly. In the case of children, parents observe that young children often have difficulty accurately describing their symptoms, complicating the monitoring of the disease's progression. As a result, pediatric upper respiratory infections are more likely to be perceived as serious conditions.

Information Search

Interviewees generally sought information only when symptoms were severe, focusing on disease diagnosis and treatment options rather than antibiotics. Their primary sources of information were medical professionals, personal experiences, and social media. However, a common concern was the perceived lack of detailed communication from doctors during consultations, which sometimes led interviewees to rely on their past experiences or social media for treatment decisions.

Medical professionals are regarded as the most credible and authoritative source of health information. However, a common concern was the perceived lack of detailed communication from doctors during consultations. They express that doctors often deliver information in a brief and non-exhaustive manner, which lacks the depth needed for full understanding. For example, a diagnosis of a viral infection might be given without a thorough explanation of its implications. Similarly, when medications are prescribed, the reasoning behind the specific choice is not always transparent. This opacity can impede interviewees' ability to comprehend the rationale behind medical decisions, making it difficult for them to assimilate accurate and comprehensive knowledge about their health condition and its management.

In situations where medical information falls short, interviewees (n=10) are inclined to fall back on treatment plans based on their own past experiences. They might assume the efficacy of certain medications based on previous encounters with similar symptoms. However, this approach, driven by a cursory understanding, can lead to misconceptions and doubts.

When evaluating the credibility of health information circulating on social media, interviewees consider the expertise of the sources and the completeness of the content. Yet, they view such information as supplementary and use it only for reference. In cases of conflicting information, the advice of medical professionals is still regarded as the ultimate benchmark for truth and

reliability.

Treatment Plan Evaluation

When evaluating treatment plans for URTIs, interviewees engage in a meticulous selection process from a range of therapeutic options. This process is structured around four key sub-themes: Evaluation Criteria, Home Treatment, Seeking Medical Treatment, and Antibiotic Treatment.

Evaluation Criteria: When evaluating treatment options for URTIs, interviewees considered factors such as the effectiveness and efficiency of medications. ' Effectiveness ' refers to the degree of symptom alleviation after ingestion, while 'efficiency' pertains to the time it takes for the medication to take effect. A small subset of interviewees (n=2) also consider medication dosage and potential side effects, weighing the perceived effectiveness of lower antibiotic dosages against other remedies and anticipating the possibility of side effects such as gastrointestinal issues or resistance, thus considering their impact to be significant.

Home Treatment: In the early or mild stages of URTI, interviewees frequently choose home-based remedies, focusing on supportive care and self-medication. Supportive care encompasses measures like dressing warmly, using blankets, taking warm baths, resting, staying hydrated, and maintaining a light diet. The rationale is to rely on the immune system to tackle less severe infections. While most interviewees recognize the palliative benefits of supportive care for mild symptoms, they also understand its limitations in more severe cases.

Depending on their initial assessment of symptom severity, interviewees may choose self-medication or explore other avenues like medical consultations. The absence of a standardized framework for treatment plan selection results in a variety of approaches for different severities of URTIs. When selecting over-the-counter medications, the majority of interviewees (n=7) depend on personal experience or advice from friends and pharmacy staff, with a small number (n=1) also consulting social media for guidance.

The choice for home treatment is largely influenced by the belief that the condition is manageable and not severe, as per the experiences of most interviewees (n=9). Additionally, some mention the high costs and inconveniences of medical treatment, including difficulties in registration, long wait times, complex procedures during the COVID-19 era, the risk of hospital-acquired infections, and financial burdens. A few (n=3) also note that self-medication reduces disruptions to their social and daily routines.

Among the 15 interviewees, 12 resorted to self-medication for their most recent URTI, using over-the-counter drugs to alleviate symptoms. The majority were able to control the progression of their condition through home treatment, with only 2 subsequently seeking

medical intervention. This highlights the preference for self-care and the perceived efficacy of home remedies in managing URTIs, while also indicating the circumstances under which medical intervention is considered necessary.

Seeking Medical Treatment: When symptoms of the last URTI appeared, 3 interviewees chose to seek medical treatment immediately, influenced by their self-assessment of the condition's severity or personal inclinations towards medical consultation. Two of these initially tried self-medication but switched to professional help due to ineffective results. All 5 who sought medical care were prescribed antibiotics; one was diagnosed with a bacterial infection via a routine blood test, while another was identified with a viral infection. However, the remaining 3 did not undergo standard blood or bacteriological tests, and doctors prescribed antibiotics with a vague diagnosis of "inflammation" without specifying the underlying cause.

Antibiotic Treatment: Regarding antibiotics, the majority of interviewees (n=8) struggle to differentiate them from other cold medications. Most (n=4) are unfamiliar with identifying common antibiotics, often mislabeling them as "anti-inflammatory drugs." Only a few (n=3) can accurately recognize them. Additionally, a small number (n=2) incorrectly assume that injectable medications are antibiotics, while oral forms are not.

In terms of the appropriate timing for antibiotic use, interviewees suggest that they would consider antibiotics as a treatment option exclusively for severe URTIs. They associate symptoms such as persistent coughing, expectoration, sore throat, and fever with "inflammation," which they believe warrant the use of antibiotics. Some interviewees advocate for the decision to use antibiotics to be made in conjunction with a medical professional.

Although 5 interviewees correctly note that antibiotics target bacterial infections, the inability to discern between viral and bacterial infections leads them to take antibiotics preemptively to manage the condition when the infection type remains unclear.

Acquisition of Antibiotics

Antibiotics were obtained through medical prescriptions, pharmacy purchases, or leftover medications. The decision to purchase antibiotics without a prescription was often driven by considerations of efficacy, cost, and convenience. Some interviewees also reported stocking up on antibiotics due to the increased difficulty of obtaining them post-COVID-19.

Use of Antibiotics

Interviewees frequently adjusted antibiotic dosages based on perceived symptom changes. A significant number (n=10) may opt to reduce the dosage, frequency of intake, or even cease the medication altogether when they perceive an improvement in symptoms. This premature discontinuation often stems from concerns regarding the side effects of antibiotics or a general

belief in the inherent risks of medication. A minority (n=2) mentioned that the fear of side effects would also lead them to adjust their dosage and frequency if no symptomatic relief is achieved within a few days of starting the medication. In contrast, two parents highlighted that they would rely on their child's doctor's advice during follow-up consultations to determine whether to continue or discontinue the antibiotic treatment.

Post-Use Evaluation

Most interviewees considered antibiotics effective for treating severe URTIs, citing rapid action and positive outcomes. These interviewees have expressed a clear intention to redeploy antibiotics for similar symptoms in the future. Despite acknowledging the potential side effects (such as liver and kidney damage, allergic reactions, and gastrointestinal disturbances) and the risk of antibiotic resistance, most had not experienced significant issues. Furthermore, despite a universal acknowledgment of antibiotic resistance, their understanding of antibiotic resistance was superficial.

Although they recognize that the misuse of antibiotics can lead to resistance, they tend to view this primarily as an issue of individual-level overuse. As a result, a significant number of interviewees (n=12) believe that their personal risk of contributing to antibiotic resistance is quite low, associating it mainly with those who frequently use antibiotics. A smaller group (n=4) has a more accurate understanding that bacterial resistance is the core issue at hand.

Only one interviewee has expressed concerns about the future implications of antibiotic resistance, suggesting that it could complicate the treatment and recovery from severe illnesses. A parent in this group also pointed out the widespread impact of antibiotic misuse in our country, noting its potential to affect the entire population. Despite these concerns, all interviewees unanimously agree that the benefits of using antibiotics to treat upper respiratory tract infections currently outweigh the associated risks and side effects. This consensus indicates that the perceived advantages of antibiotics are not easily overshadowed by concerns about resistance, and interviewees are not inclined to change their approach based on these considerations.

Impact of the Epidemic

Interviewees demonstrate a keen awareness of COVID-19, recognizing it as a viral infection. However, this understanding has not notably shifted their perception of URTIs. A group (n=9) sees COVID-19 symptoms as akin to the common cold but asserts the ability to differentiate between the two based on symptom analysis, travel history, and personal protection measures, expressing confidence in this discernment. Given the irreversible damage associated with COVID-19, its high infectivity, and the disruptions it causes to social and professional life,

these interviewees (n=9) consider it a graver disease. This has heightened their vigilance towards respiratory symptoms and prompted proactive self-protection measures, with some (n=7) noting a marked reduction in the frequency of such symptoms due to effective precautions.

Most interviewees said that their COVID-19 experiences have not substantially altered their treatment behaviors for upper respiratory tract infections. Yet, the pandemic's impact on healthcare processes has introduced complexities, causing inconvenience. For instance, those presenting fever symptoms are now required to visit fever clinics. The altered medical landscape and the risk of cross-infection in hospitals have led some (n=8) to prefer home treatment. Conversely, a minority (n=1) appreciates the improved order, enhancement, and safety of medical processes, making them more inclined to seek professional care. Additionally, the stricter regulations on purchasing medication, including requirements for personal information registration and restrictions on over-the-counter cold remedies, have prompted some (n=4) to stock up on medications to facilitate self-medication.

Conclusion

The study reveals that the trajectory from the identification of needs to the post-use evaluation of antibiotics constitutes a self-reinforcing cycle. This cycle tends to escalate pepole's demand for antibiotic use, particularly following their application in treating URTIs, thereby fostering potentially irrational antibiotic consumption patterns. Within this cycle, the identification of needs, the assessment of alternative treatment options, and the acquisition process of antibiotics emerge as pivotal elements that directly influence the parent's therapeutic choices for URTIs.

During the need identification phase, individuals typically gauge the severity of their URTIs by the severity and duration of symptoms, subsequently selecting treatment plans that align with their assessed condition. In the alternative evaluation phase, parents categorize medications into "potent" or "milder" classes based on their efficacy and the time to take effect, tailoring their treatment approach to the perceived severity of their ailment. For mild infections, milder drugs are deemed sufficient; whereas, severe cases are addressed with more potent medications.

In addition, the public also evaluates the efficacy of drugs based on the side effects or resistance after drug use. For antibiotics, the public generally believes that they are potent drugs used to treat severe URTIs. At the stage of obtaining drugs, the public will consider the input during the process of obtaining antibiotics, including time and total cost. Due to considerations of price and accessibility, the behavior of buying drugs without a prescription

at the pharmacy is quite common.

Finally, seven attributes were determined: severity of symptoms, duration, antibiotic effect, time to take effect, risk of side effects and antibiotic resistance, total cost, and time spent. The attributes and their respective levels, as determined by this study, are detailed in the accompanying Table S1-8.

[Table S1-8]

Table S1-1 Literature Search Strategy

Database	Search Strategy
Pubmed	(("outpatient*"[Title/Abstract] OR "the public"[Title/Abstract] OR "people*"[Title/Abstract] OR "population*"[Title/Abstract] OR "consumer*"[Title/Abstract] OR "customer*"[Title/Abstract] OR "user*"[Title/Abstract] OR "purchaser*"[Title/Abstract] OR "citizen*"[Title/Abstract] OR "resident*"[Title/Abstract] OR "inhabitant*"[Title/Abstract] OR ("outpatients"[MeSH Terms] OR ("population"[MeSH Terms] OR "population groups"[MeSH Terms]) OR "population groups"[MeSH Terms])) AND (("knowledge*"[Title/Abstract] OR "aware*"[Title/Abstract] OR "understand*"[Title/Abstract] OR "attitude*"[Title/Abstract] OR "view*"[Title/Abstract] OR "percept*"[Title/Abstract] OR "perceiv*"[Title/Abstract] OR "opinion*"[Title/Abstract] OR "belie*"[Title/Abstract] OR "concern*"[Title/Abstract] OR "fear*"[Title/Abstract] OR "accept*"[Title/Abstract] OR "perspect*"[Title/Abstract] OR "worr*"[Title/Abstract] OR "concep*"[Title/Abstract] OR "determina*"[Title/Abstract] OR "stimulus*"[Title/Abstract] OR "incent*"[Title/Abstract] OR "reason*"[Title/Abstract] OR "motiv*"[Title/Abstract] OR "rationa*"[Title/Abstract] OR ("health knowledge, attitudes, practice"[MeSH Terms] OR "patient medication knowledge"[MeSH Terms] OR "awareness"[MeSH Terms] OR "comprehension"[MeSH Terms] OR "attitude"[MeSH Terms] OR "attitude to health"[MeSH Terms] OR "culture"[MeSH Terms] OR "health belief model"[MeSH Terms] OR "fear"[MeSH Terms] OR "concept formation"[MeSH Terms] OR "generalization, stimulus"[MeSH Terms] OR "motivation"[MeSH Terms])) AND ("anti bacterial agents"[MeSH Terms] OR "anti infective agents"[MeSH Terms] OR ("antibiotic*"[Title/Abstract] OR "antimicro*"[Title/Abstract] OR "antibact*"[Title/Abstract] OR "anti infect*"[Title/Abstract])) AND ("respiratory tract infection*"[Title/Abstract] OR "respiratory infection*"[Title/Abstract] OR "upper respiratory infection*"[Title/Abstract] OR "upper respiratory tract infection*"[Title/Abstract] OR ("cold*"[Title/Abstract] OR "rhinitis*"[Title/Abstract] OR "cough*"[Title/Abstract] OR "sore throat*"[Title/Abstract] OR "pharyngitis*"[Title/Abstract] OR "flu"[Title/Abstract] OR "influenza*"[Title/Abstract]) OR ("runny nose*"[Title/Abstract] OR "nasal congestion"[Title/Abstract] OR "sneez*"[Title/Abstract]) OR ("respiratory tract infections"[MeSH Terms] OR "pharyngitis"[MeSH Terms] OR "common cold"[MeSH Terms] OR "rhinitis"[MeSH Terms] OR "influenza, human"[MeSH Terms] OR "rhinorrhea"[MeSH Terms] OR "sneezing"[MeSH Terms] OR "cough"[MeSH Terms])))) AND (english)
Embase	antibiotic OR antimicrobial OR antibacterial in Title Abstract Keyword AND public OR outpatient OR population OR people OR customer OR user OR resident in Title Abstract Keyword AND respiratory tract infection OR common cold OR pharyngitis OR rhinitis OR cough OR influenza OR rhinorrhea OR sneezing OR sore throat OR runny nose OR nasal congestion in Title Abstract Keyword AND knowledge OR attitude OR practice OR behvior in Title Abstract Keyword - (Word variations have been searched)
Web of Science	TI=(knowledge* OR aware* OR understand* OR attitude* OR view* OR perception* OR perceiv* OR opinion* OR belie* OR concern* OR fear* OR accept* OR perspect* OR worr* OR concept* OR determina* OR stimulus* OR incent* OR reason* OR motiv* OR rationa*) OR A B=(knowledge* OR aware* OR understand* OR attitude* OR view* OR perception* OR perceiv* OR opinion* OR belie* OR concern* OR f ear* OR accept* OR perspect* OR worr* OR concept* OR determina* OR stimulus* OR incent* OR reason* OR motiv* OR rationa*) AND TI=(Antibiotic* OR antimicro* OR antibact* OR antiinfec*) OR AB=(Antibiotic* OR antimicro* OR antibact* OR antiinfec*)<br>AND<br>TI=(outpatient* OR "the public" OR people* OR population* OR consumer* OR customer* OR user* OR purchaser* OR citizen* OR residen t* OR inhabitant*) OR AB=(outpatient* OR "the public" OR people* OR population* OR consumer* OR customer* OR user* OR purchaser* OR citizen* OR resident* OR inhabitant*) AND<br>TI=(respiratory tract infection* OR respiratory infection* OR upper respiratory tract infection* OR upper respiratory infection* OR cold* OR

	rhinitis* OR cough* OR sore throat* OR pharyngitis* OR flu* OR influenza* OR runny nose* OR nasal congestion OR sneez*) OR AB=(resp iratory tract infection* OR respiratory infection* OR upper respiratory tract infection* OR upper respiratory infection* OR cold* OR rhinitis* OR cough* OR sore throat* OR pharyngitis* OR flu* OR influenza* OR runny nose* OR nasal congestion OR sneez*)
Cochrane Library	1. (antibiotic or antimicrobial or antibacteria).mp. [mp=title, abstract, heading word, drug trade name, original title, device manufacturer, drug manufacturer, device trade name, keyword heading word, floating subheading word, candidate term word]<br>2. (outpatient or people or population or consumer or customer or purchaser or citizen or resident or inhabitiant or user or public).mp. [mp=title, abstract, heading word, drug trade name, original title, device manufacturer, drug manufacturer, device trade name, keyword heading word, floating subheading word, candidate term word]<br>3. (respiratory tract infection or upper respiratory infection or cold or rhinitis or cough sore throat or pharyngitis or flu or influenza or runny nose or nasal congestion or sneezing).mp. [mp=title, abstract, heading word, drug trade name, original title, device manufacturer, drug manufacturer, device trade name, keyword heading word, floating subheading word, candidate term word]<br>4. (knowledge or awareness or understanding or attitude or view or perception or perceive or opinion or belief or concern or fear or acceptance or perception or worry or concept or determinant or stimulus or incentive or reason or motives or ratinale).mp. [mp=title, abstract, heading word, drug trade name, original title, device manufacturer, drug manufacturer, device trade name, keyword heading word, floating subheading word, candidate term word]<br>5. 1 and 2 and 3 and 4

Table S1-2 Public Medical Seeking Behavior for Upper Respiratory Tract Infections and Influencing Factors

Study	Behavior		Influencing Factors		
	Measurement Indicator	Frequency (%)	Disease-Related	Antibiotics-Related	Personal characteristic
Roope, L.S.J. et al (2018)	Adults' medical seeking behavior for influenza-like illness (simulated)	47.90			
Luque, J.S. et al (2008)	Parents' medical seeking behavior for different combinations of upper respiratory tract infection symptoms	67.00 - 83.50			Bicycle ownership (↓)<br>Non-dirt floor (↑)<br>Television (↑)<br>High income (↑)
Chai, J. et al (2019)	Adults' medical seeking behavior for respiratory symptoms within 12 months	59.30		Lack of knowledge about antibiotics (↑)	Having medical insurance<br>High level of education (↓)
Andre, M. et al (2007)	Parents' medical seeking behavior for 18-month-old children's respiratory infections	22.90	Higher degree of concern for upper respiratory symptoms (↑)		
Carling, C.L. et al (2009)	Adults' medical seeking behavior for sore throat (simulated)	22.70	Improved understanding of the causes and treatment measures for sore throat (↓)<br>Lower perceived importance of sore throat (↓)		
Lin, L. et al (2020)	College students' medical seeking behavior for upper respiratory symptoms within a month	25.10	Presence of fever symptoms (↑)<br>Presence of multiple symptoms (↑)	History of storing antibiotics (↑)<br>History of purchasing antibiotics without a prescription (↑)<br>Higher perceived severity of upper respiratory symptoms (↑)<br>Perceived effectiveness of antibiotics (↑)<br>High awareness of antimicrobial resistance (AMR) (↓)	Medical background (↓)
Lin, L. et al (2021)	Parents' medical seeking behavior for children's upper respiratory symptoms within a month	68.60	Higher perceived severity of upper respiratory symptoms (↑)<br>Presence of fever symptoms (↑)	History of storing antibiotics (↓)	Parents with a medical background (↓)

				Perceived effectiveness of antibiotics (↑)<br>Source of information (△)	
Cheng, J., et al (2019)	Parents' medical seeking behavior for children's upper respiratory symptoms within a year	78.00	Presence of specific symptoms (↑)	History of storing antibiotics (↓)	
Emslie, M.J. et al (2003)	Adults' medical seeking behavior for upper respiratory symptoms in the past 5 months	23.00	Different upper respiratory tract infection (URTI) symptoms (△)<br>High perception of the severity of URTI symptoms (↑)		
Mainous, A.G., 3rd,et al (1997)	Adults' medical seeking behavior when experiencing sore throat, cough, and clear nasal discharge for 5 days (simulated)	42.00			High education level (↓)<br>High family income (↓)<br>Medical insurance (△)
	Adults' medical seeking behavior when experiencing sore throat, cough, and colored nasal discharge for 5 days (simulated)	72.00			Medical insurance (△)
	Adults' medical seeking behavior due to sore throat, cough, and clear nasal discharge within a year	31.00			
	Adults' medical seeking behavior due to sore throat, cough, and colored nasal discharge within a year	35.00			
You, J.H. et al. (2008)	Adults' most recent medical seeking behavior for upper respiratory symptoms	92.50			
Freidoony, L. et al (2017)	Adults' medical seeking behavior for upper respiratory symptoms within 6 months	59.30	High perception of the severity of URTI symptoms (↑)	Perception of the effectiveness of antibiotics (↑)	Medical insurance (△)
Friedman, J.F. et al (2003)	Parents' medical seeking behavior for upper respiratory symptoms in children	N/A	High awareness of URTI and antibiotic use (↓)		Parents' income (Only for URTI symptoms with green nasal discharge) (↑)

Verica Ivanovska., et al (2013)	Adults' medical seeking behavior for upper respiratory tract infection (URTI) symptoms within 6 months	32.90			
	Parents' medical seeking behavior for URTI symptoms in children under 5 years old within 6 months	94.50			
A F Y Tang., et al (2004)	Adults' early medical seeking behavior for URTI symptoms (within 2 days of symptom onset)	46.90	History of URTI (↓)<br>Presence of fever symptoms (↑)		Social status (△)<br>Unemployment (↑)
	Parents' early medical seeking behavior for their children's URTI symptoms (within 2 days of symptom onset)	45.10	Presence of fever symptoms (↑)<br>High perception of the severity of URTI symptoms (↑)<br>Belief in the necessity of seeing a doctor for a cold (↑)		
D. Osborne and H.Sinclair (2006)	Patients' medical seeking behavior for respiratory infection symptoms within the past 5 months	14.10			
L. S. J. Roope, et al (2020)	Changes in adults' medical seeking behavior for simulated influenza-like illness	29.10-46.10 (Less likely/less likely to seek medical care)<br><br>10.30-14.10 (More likely/more likely to seek medical care)		Received information about antimicrobial resistance (△)<br>Shocked by provided information on antibiotic resistance (↑)	Perception of the effectiveness of antibiotics (↑)
	Changes in adults' medical seeking behavior for simulated influenza-like illness in children	N/A		Received information about antimicrobial resistance (△)<br>Shocked by provided information on antibiotic resistance (↑)	Perception of the effectiveness of antibiotics (↑)

Please note that the symbols (↑) and (↓) denote positive and negative correlations, respectively, and (△) indicates a relevant relationship. The term "N/A" would be used if the data is not available, and blank cells indicate no significant factors found.

Table S1-3 Public Expectations for Antibiotic Use in Upper Respiratory Tract Infections and Influencing Factors

Study	Behavior		Influencing Factors		
	Measurement indicator	Frequency (%)	Disease-Related	Antibiotics-Related	Personal characteristic
Braun, B.L. et al (2000)	Adults' expectations for antibiotic use for cold symptoms	49.60	High perception of the severity of upper respiratory tract infection (URTI) symptoms (↑)<br>Perception of long duration of URTI symptoms (↑)<br>Confidence in treating the cold (↑)	Belief that many people use antibiotics to treat the cold (↓)<br>Perception of the effectiveness of prescription drugs (↑)	
	Parents' expectations for antibiotic use for their children's cold symptoms	30.10	High perception of the severity of URTI symptoms (↑)<br>Desire to alleviate symptoms (↑)	Perception of the effectiveness of antibiotics (↑)	
Roope, L.S.J. et al (2018)	Adults' expectations for antibiotic use for 5-day flu-like illness	38.90		Perception of the effectiveness of antibiotics (↑)<br>Shocked by provided information on antibiotic resistance (↑)	Future low discount rate (↓)
Broniatowski, D.A. et al (2018)	Adults' expectations for antibiotic use for the last episode of upper respiratory tract infection symptoms	N/A		Perception of the effectiveness of antibiotics (↑)<br>Attitude towards the effectiveness of antibiotics (↑)	
Lin, L. et al (2020)	College students' demand for antibiotics for upper respiratory tract infection symptoms within 1 month	17.30			
Lin, L. et al (2021)	Parents' demand for antibiotics for their children's upper respiratory tract infection symptoms within 1 month	7.70	High perception of the severity of URTI symptoms (↑)<br>Presence of fever (↑)	History of storing antibiotics (↑)<br>High ability of parents to identify antibiotics (↑)<br>Perception of the effectiveness of antibiotics (↑)	
McNulty, C. A., et al (2013)	Adults' expectations for antibiotic use for upper respiratory tract infection symptoms within 6 months	53.10			
Goggin, K.et al (2020)	Parents' expectations for antibiotics for their children's	28.30		Parents' lack of knowledge about antibiotics (↑)	Parents' background (non-English) (↑)

	acute respiratory infection symptoms			Improvement in parents' knowledge about antibiotic use (↓)	Young parents (↑)<br>Low education level (↑)
Emslie, M.J. et al (2003)	Adults' expectations for antibiotics for different upper respiratory tract infection symptoms	0.30-67.20	Different URTI symptoms (△)		
Gaarslev, C.,et al (2016)	Adults' expectations for antibiotics for colds or flu	19.50		Lack of knowledge about antibiotics (↑)<br>Lack of awareness about antibiotic resistance (↑)	Parents' background (non-English) (↑)<br>Younger age (↑)
	Adults' simulated demand for antibiotics for colds or flu	16.90		Lack of knowledge about antibiotics (↑)	Parents' background (non-English) (↑)<br>Younger age (↑)<br>Higher education (↑)
Faber, M.S.,et al (2010)	Adults' expectations for antibiotics for cold symptoms	10.50	Presence of upper respiratory tract infection symptoms (cough, cold, sore throat, and fever)	(↑) - Past usage experience (↑)<br>Perception of the effectiveness of antibiotics (↑)<br>Lack of awareness about antibiotic resistance (↑)<br>Lack of knowledge about antibiotics (↑)	Higher education (↓)
	Adults' expectations for antibiotics for influenza	46.90			
	Adults' expectations for antibiotics for pneumonia	92.70			
Davis, M.E.,et al (2017)	Adults' expectations for antibiotics for coughs or the common cold	22.00		Lack of knowledge about antibiotics (↑)	Younger age (↑)
Kong, L.S.,et al (2019)	Elderly's expectations for antibiotics for different URTI symptoms	27.90-55.70		Lack of knowledge about antibiotics (↑)<br>Perception of the effectiveness of antibiotics (Specifically for colds, flu, and coughs) (↑)	
Faidah, H.S.,et al (2019)	Parents' expectations for antibiotics for children's URTI symptoms	53.00	Different upper respiratory tract infection symptoms (△)		

El Khoury, G.,et al (2017)	Parents' expectations for antibiotics for children's URTI symptoms	15.70			
Hernández-Díaz, I. et al (2019)	Caregivers' expectations for antibiotics for children's URTI symptoms	14.40			
Freidoony, L. et al (2017)	Adults' expectations for antibiotics for URTIs within 6 months	14.20			
Mangione-Smith, R. et al (2004)	Parents' expectations for antibiotics for children's URTI symptoms	70.00	Presence of ear pain (↑)<br>High level of concern for upper respiratory tract infections (↑)		Parental background (non-Hispanic white) (↑)
Parimi, N. et al (2004)	Caregivers' demands for antibiotics for children's URTI symptoms	22.60	Lack of awareness (↑)		
Hong, J.S. et al	Patients' expectations for antibiotics for URTIs	50.00		Experience with using antibiotics for upper respiratory symptoms (↑)	
Nasser M., et al (2020)	Demand for antibiotics to treat URTIs during medical visits	59.70		Physician characteristics (△)	Adult patients (compared to children) (↑)<br>Education level of college and below (↑)
Slawomir Chlabicz., et al (2019)	Demand for doctors to use antibiotics to treat URTIs after intervention	60.20		Medical institutions providing physician intervention measures and patient education (↑)	Education level below secondary vocational (compared to college and undergraduate education) (↑)
	Patients' demand for antibiotics for URTIs	16.70			
	Patients' demand for non-antibiotic treatments for URTIs	5.50	Presence of different symptoms (△)		
E. A.Belongia., et al (2002)	Adults' demand for antibiotics to treat URTIs within 6 months	27.80			

	Parents' demand for antibiotics to treat children's URTIs within 6 months	15.20			
D. Osborne. et.al (2006)	Patients' demands for antibiotic treatment for URTI symptoms	0.40 - 66.50			
L. S. J. Roope, et al (2020)	Parents' changing expectations for antibiotics for simulated children's flu-like illnesses			Received information about antibiotic resistance (△) Shocked by provided information on antibiotic resistance (↑)<br>Perception of the effectiveness of antibiotics (↑)	
	Adults' changing expectations for antibiotics for simulated flu-like illnesses	42.30-54.70 (Less likely)<br>7.50-10.10 (More likely)		Perception of the effectiveness of antibiotics (↑)	

Please note that the symbols (↑) and (↓) denote positive and negative correlations, respectively, and (△) indicates a relevant relationship. The term "N/A" would be used if the data is not available, and blank cells indicate no significant factors found.

Table S1-4 Public Self-Medication Behavior with Antibiotics for Upper Respiratory Tract Infections and Influencing Factors

Study	Behavior		Influencing Factors		
	Measurement Indicator	Frequency (%)	Disease-Related	Antibiotics-Related	Personal characteristic
Chai, J. et al (2019)	Adults purchasing over-the-counter antibiotics to treat respiratory infections within 12 months	15.92			
	Adults using leftover antibiotics to treat respiratory infections within 12 months	13.10			
Lin, L. et al (2020)	College students using antibiotics to treat upper respiratory tract infection (URTI) symptoms within a month	16.30	Presence of multiple symptoms (↑)<br>High perception of the severity of upper respiratory tract infection symptoms (↑)	Perception of the effectiveness of antibiotics (↑)<br>History of storing antibiotics (↑)<br>History of purchasing antibiotics without a prescription (↑)	Place of residence (△)

	College students using over-the-counter antibiotics to treat URTI symptoms within a month	12.50			
	College students using leftover antibiotics to treat URTI symptoms within a month	10.20	Source of information (△)		
Lin, L. et al (2021)	Parents using antibiotics to treat URTI symptoms within a month	18.60	High perception of the severity of upper respiratory tract infection symptoms (↑)<br>Presence of fever symptoms	(↑) - Higher ability of parents to identify antibiotics (↑)<br>History of storing antibiotics (↑)	
Cheng, J., et al (2019)	Parents using antibiotics to treat children's URTI symptoms within a year	20.50	Presence of obvious nasal discharge (↑)	History of storing antibiotics (↑)	Older parents (↑)<br>Larger family size (↑)
McNulty, C. A. , et al (2013)	Adults using leftover antibiotics to treat URTI symptoms within the past 6 months	0.40			
El Khoury, G. , et al (2017)	Parents using over-the-counter antibiotics to treat their children's URTI symptoms	5.20			
	Parents medicating their children's fever symptoms with antibiotics	6.50			
You, J.H. et al (2008)	Adults using over-the-counter antibiotics to treat the most recent episode of URTI symptoms	7.30			
You, J.H. et al (2008)	Adults using antibiotics from family or friends to treat the most recent episode of URTI symptoms	1.10			
Hernández-Díaz, I. et al (2019)	Parents using over-the-counter antibiotics to treat their children's URTI symptoms	<0.01			
	Parents using antibiotics to treat their children's fever symptom	6.20			
Freidoony, L. et al (2017)	Adults using leftover antibiotics within 6 months	3.50			
Ngu, R.C. et al （2018)	Adults self-medicating with antibiotics before seeking medical treatment for respiratory infections	41.90			History of tuberculosis (↓)

Grigoryan, L. et al (2007)	Adults using leftover antibiotics within 12 months	N/A		Experience of treating upper respiratory tract infection symptoms without a prescription (↑)	Place of residence (△)
Parimi, N. et al (2004)	Caregivers self-medicating children's URTI symptoms with antibiotics within 30 days	33.10	High perception of the severity of upper respiratory tract infection symptoms (↑)		
	Caregivers using over-the-counter antibiotics to treat children's URTI symptoms	28.00	Presence of different symptoms (△)		
Landers, T.F. et al (2010)	Adults and parents self-medicating themselves or their children with antibiotics for URTI	23.60		Higher ability to recognize non-antibiotic drug treatment for URTIs (↓)	
Togoobaatar, G. et al (2010)	Caregivers self-medicating children with antibiotics for URTIs within 6 months	42.30		History of storing antibiotics (↑)<br>Mothers using antibiotics for self-medication (↑)<br>Mothers with higher knowledge of antibiotics (↓)<br>Trend of expecting antibiotics (↑)	Increase in children's age (↑)
Verica Ivanovska., et al (2013)	Adults using antibiotics to treat URTI symptoms within 6 months	17.80			
	Adults using over-the-counter antibiotics to treat URTI symptoms within 6 months	6.00			
Verica Ivanovska., et al (2013)	Adults using leftover or relatives' antibiotics to treat URTI symptoms within 6 months	11.80			
Verica Ivanovska., et al (2013)	Parents using over-the-counter antibiotics to treat URTI symptoms in children under 5 years old	<0.01			
	Parents using leftover or relatives' antibiotics to treat URTI symptoms within 6 months	1.80			

Tan Y S L., et al (2006)	Adults self-medicating with antibiotics before seeking medical treatment for URTI symptoms	4.90			
Slawomir Chlabicz., et al (2019)	Patients self-medicating with antibiotics before seeking medical treatment for URTI symptoms	7.60			
S. M. Hussain, et al (2020)	Parents using antibiotics to treat their children's fever symptoms	15.70			
Y. Luo, et al (2021)	Adults using antibiotics to treat cough symptoms within the past 12 months	12.20		Past experience with antibiotics (↓)<br>Moderate level of antibiotic awareness (lower level) (↑)	Age (△)<br>Presence of chronic diseases (↓)

Please note that the symbols (↑) and (↓) denote positive and negative correlations, respectively, and (△) indicates a relevant relationship. The term "N/A" would be used if the data is not available, and blank cells indicate no significant factors found.

Table S1-5 Public Compliance Behavior in the Use of Antibiotics for Upper Respiratory Tract Infections and Influencing Factors

Study	Behavior		Influencing Factors		
	Measurement Indicator	Frequency (%)	Disease-Related	Antibiotics-Related	Personal characteristic
Perez-Gorricho, B. et al (2003)	Adult compliance with antibiotic use for respiratory tract infections	86.00 (Daily Dose Compliance) 85.00 (Treatment Duration Compliance)		Mode of antibiotic administration (△)	
Perez-Gorricho, B. et al (2003)	Adult compliance with prescribed antibiotics for respiratory tract infections	86.00 (Daily Dose Compliance) 85.00 (Treatment Duration Compliance)			
McNulty, C. A., et al (2013)	Adult compliance with prescribed antibiotics for respiratory tract infections within 6 months	74.30			
El Khoury, G.et al (2017)	Parents' compliance with prescribed antibiotics for children's respiratory tract infections	80.40 (Daily Dose Compliance) 89.20 (Treatment Duration Compliance)			
You, J.H. et al (2008)	Adult compliance with prescribed antibiotics for upper respiratory tract infections	78.50			
Hernández-Díaz, I. et al (2019)	Caregivers' compliance with antibiotic treatment for children's respiratory tract infections	72.20(Daily Dose Compliance) 83.50 (Treatment Duration Compliance)			Higher education level (↑)
Freidoony, L. et al (2017)	Adult compliance with antibiotic use for respiratory tract infections within 6 months	64.30			
Parimi, N. et al (2004)	Caregivers' compliance with antibiotic treatment for children's respiratory tract infections	67.70			
Pechere, J.C. et al (2001)	Caregivers' compliance with antibiotic treatment for themselves or their children's respiratory tract infections	69.00			

Please note that the symbols (↑) and (↓) denote positive and negative correlations, respectively, and (△) indicates a relevant relationship. The term "N/A" would be used if the data is not available, and blank cells indicate no significant factors found

Table S1-6 Public Preferences Attribute Pool for the Irrational Use of Antibiotics

Order	Attribute	Description	Quantitative Review	Qualitative Review
1	Disease Severity	Mild, moderate, or severe symptoms	√	√
2	Duration	Number of days symptoms have been present since onset	√	√
3	Specific Symptom Occurrence	Presence of specific symptoms, such as fever	√	√
4	Disease Awareness	Understanding of the disease, such as the probability of bacterial infection	√	
5	Antibiotic Effectiveness	Reduction in the intensity of symptoms	√	√
6	Time to Antibiotic Effect	Time until the antibiotic starts to take effect after being taken		√
7	Antibiotic Side Effects or Resistance Risk	No risk, low risk, moderate risk, high risk	√	√
8	Antibiotic Storage	Condition Whether antibiotics are stored	√	√
9	Past Antibiotic Treatment Experience	Effectiveness and ineffectiveness of past antibiotic treatments	√	√
10	Source of Antibiotic Use Recommendation	Doctor, family, friends	√	√
11	Source of Antibiotics	Doctor, nurse, pharmacy, leftover prescriptions, etc.	√	
12	Time Required to Obtain Antibiotics	Total time for travel and consultation to obtain antibiotics		√
13	Total Cost of the Antibiotic Acquisition Process	Total cost of travel, consultation, and other expenses in the process of obtaining antibiotics		√
14	Waiting Time for Treatment	Waiting time for treatment measures		√

Table S1-7 Thematic Analysis Results of Interview Data

Theme	Theme Description	Sub-themes
Need Identification	Early individual recognition and assessment when symptoms of upper respiratory tract infection occur	- Symptom Discovery<br>-Disease Attribution<br>-Disease Identification
Information Search	Behavior of searching for information related to upper respiratory tract infection symptoms and treatment plans	- Doctor<br>-Personal Experience<br>- Others
Treatment Plan Evaluation	Individual evaluation and selection process for different treatment plans for upper respiratory tract infection	-Evaluation Criteria<br>- Home Treatment<br>- Seeking Medical Treatment<br>-Antibiotic Treatment
Acquisition of Antibiotics	Channels through which individuals obtain antibiotics	_
antibiotic Use	Individual behavior of taking antibiotics as prescribed by a doctor	_
Post-Use Evaluation	Individual evaluation of the effectiveness after using antibiotics	-Positive Evaluation<br>-Negative Evaluation
Impact of the Epidemic	Changes in individual evaluation and treatment behavior for upper respiratory tract infection in the context of the COVID-19 pandemic	-Impact on Disease Perception<br>-Impact on Treatment Measures

Table S1-8 Attributes and levels of antibiotic use behavior in the discrete choice experiment

No.	Attribute	Level	Definition
1	Symptoms	Mild/Moderate/Severe	Indicates the severity of symptoms
2	Duration	1 day/3 days/5 days/7 days	Indicates the number of days symptoms persist, i.e., the time from the onset of symptoms
3	Time to effect after using antibiotics	2 hours/4 hours/8 hours	Indicates the time it takes for antibiotics to take effect after being taken. The shorter the time, the faster the antibiotic works.
4	Antibiotic effect	Half recovered (50%) / Basically recovered (90%)	Indicates the degree of symptom relief after taking antibiotics, the higher the probability, the better the antibiotic's effect on relieving symptoms
5	Risk of Side Effects or Resistance	No risk (0%) / Low risk (10%) / Moderate risk (50%) / High risk (90%)	Indicates the likelihood of experiencing adverse reactions or the development of antibiotic resistance after taking antibiotics. The higher the probability, the greater the risk of this outcome.
6	Time to Obtain Antibiotics	0.5 hours / 2 hours / 4 hours / 6 hours	The time spent in obtaining antibiotics.
7	Total Cost	50 yuan / 150 yuan / 250 yuan / 400 yuan	The total cost incurred in the process of obtaining antibiotics, including transportation fees, the cost of purchasing medication, or fees generated during the medical consultation process.

References:

1. Braun V, Clarke V. Using thematic analysis in psychology. Qualitative Research in Psychology. 2006;3(2):77-101. doi: 10.1191/1478088706qp063oa.
2. Helter TM, Boehler CE. Developing attributes for discrete choice experiments in health: a systematic literature review and case study of alcohol misuse interventions. Journal of Substance Use. 2016;21(6):662-8. Epub 2016/10/04. doi: 10.3109/14659891.2015.1118563. PubMed PMID: 27695386; PubMed Central PMCID: PMCPMC5022136.

Appendix S2 Questionnaire on the Use of Antibiotics by Parents of Children with Colds

Dear Sir/Madam,

Hello! We are graduate students from Tongji Medical College of Huazhong University of Science and Technology. In order to improve the rational treatment of common colds among the public, we are conducting a research study on the public treatment patterns for the common cold. The survey data will be used for academic research purposes only, and we will strictly protect the confidentiality of your information. Please read the following instructions carefully before answering the questionnaire. **There are no correct answers in this questionnaire, please answer according to your actual situation.** The questionnaire will take approximately **10-15 minutes** to complete. Thank you for your participation!

Tongji Medical College, Huazhong University of Science and Technology

Instructions

Antibiotics are a class of drugs, and common antibiotics include: "Penicillin" such as amoxicillin, "Cephalosporin" such as cefadroxil, "Quinolone" such as norfloxacin and levofloxacin, "Macrolide" such as penicillin and azithromycin, "Tetracycline" such as doxycycline. All of these belong to antibiotics.

Part 1: Survey on Antibiotic Usage Decision-Making by Parents of Children with Colds (Block 1)

1. This part consists of 9 scenarios, each with two situations. This section focuses on how you weigh the decision-making process of using antibiotics when your child has a cold. There are no correct answers for each question. Based on your experience, please assess the characteristics of Scenario A and Scenario B and decide whether to use antibiotics for your child. Please tick only one box for each question.

Choice set 1:	Scenario A	Scenario B
Symptoms	Moderate	Severe
Duration	5 days	1 day
The onset time of antibiotic	2 hours	4 hours
Intensity of symptom relief after antibiotics	50%	90%
Risk of side effects or antibiotic resistance	High risk (90%)	Low risk（10%）
Time required to obtain antibiotics	6 hours	2 hours
Total cost	400 yuan	150 yuan

①In which situation am I more inclined to use antibiotics for my child?

☐ Scenario A ☐ Scenario B ☐ Neither

②In which situation am I more likely to directly purchase antibiotics for my child from a pharmacy?

☐ Scenario A ☐ Scenario B ☐ Neither

Choice set 2:	Scenario A	Scenario B
Symptoms	Mild	Severe
Duration	7 days	3 days
The onset time of antibiotic	2 hours	8 hours
Intensity of symptom relief after antibiotics	90%	50%
Risk of side effects or antibiotic resistance	Low risk (10%)	No risk（0%）
Time required to obtain antibiotics	0.5 hours	6 hours
Total cost	150 yuan	250 yuan

①In which situation am I more inclined to use antibiotics for my child?

☐ Scenario A ☐ Scenario B ☐ Neither

②In which situation am I more likely to directly purchase antibiotics for my child from a pharmacy?

☐ Scenario A ☐ Scenario B ☐ Neither

Choice set 3:	Scenario A	Scenario B
Symptoms	Severe	Mild
Duration	7 days	3 days
The onset time of antibiotic	4 hours	2 hours
Intensity of symptom relief after antibiotics	50%	90%
Risk of side effects or antibiotic resistance	Moderate risk (50%)	High risk（90%）
Time required to obtain antibiotics	2 hours	4 hours
Total cost	50 yuan	150 yuan

①In which situation am I more inclined to use antibiotics for my child?

☐ Scenario A ☐ Scenario B ☐ Neither

②In which situation am I more likely to directly purchase antibiotics for my child from a pharmacy?

☐ Scenario A ☐ Scenario B ☐ Neither

Choice set 4:	Scenario A	Scenario B
Symptoms	Mild	Moderate
Duration	1 days	3 days
The onset time of antibiotic	8 hours	4 hours
Intensity of symptom relief after antibiotics	50%	90%
Risk of side effects or antibiotic resistance	Low risk（10%）	Moderate risk (50%)
Time required to obtain antibiotics	6 hours	0.5 hours
Total cost	400 yuan	50 yuan

①In which situation am I more inclined to use antibiotics for my child?

☐ Scenario A ☐ Scenario B ☐ Neither

②In which situation am I more likely to directly purchase antibiotics for my child from a pharmacy?

☐ Scenario A ☐ Scenario B ☐ Neither

Choice set 5:	Scenario A	Scenario B
Symptoms	Moderate	Severe
Duration	5 days	3 days
The onset time of antibiotic	2 hours	4 hours
Intensity of symptom relief after antibiotics	90%	50%
Risk of side effects or antibiotic resistance	No risk（0%）	Moderate risk (50%)
Time required to obtain antibiotics	4 hours	0.5 hours
Total cost	250 yuan	400 yuan

①In which situation am I more inclined to use antibiotics for my child?

☐ Scenario A ☐ Scenario B ☐ Neither

②In which situation am I more likely to directly purchase antibiotics for my child from a pharmacy?

☐ Scenario A ☐ Scenario B ☐ Neither

Choice set 6:	Scenario A	Scenario B
Symptoms	Severe	Mild
Duration	5 days	7 days
The onset time of antibiotic	4 hours	8 hours
Intensity of symptom relief after antibiotics	90%	50%
Risk of side effects or antibiotic resistance	High risk（90%）	Moderate risk (50%)
Time required to obtain antibiotics	0.5 hours	4 hours
Total cost	50 yuan	250 yuan

①In which situation am I more inclined to use antibiotics for my child?

☐ Scenario A ☐ Scenario B ☐ Neither

②In which situation am I more likely to directly purchase antibiotics for my child from a pharmacy?

☐ Scenario A ☐ Scenario B ☐ Neither

Choice set 7:	Scenario A	Scenario B
Symptoms	Moderate	Mild
Duration	1 days	5 days
The onset time of antibiotic	8 hours	4 hours
Intensity of symptom relief after antibiotics	90%	50%
Risk of side effects or antibiotic resistance	Moderate risk (50%)	No risk（0%）
Time required to obtain antibiotics	4 hours	6 hours
Total cost	50 yuan	150 yuan

①In which situation am I more inclined to use antibiotics for my child?

☐ Scenario A ☐ Scenario B ☐ Neither

②In which situation am I more likely to directly purchase antibiotics for my child from a pharmacy?

☐ Scenario A ☐ Scenario B ☐ Neither

Choice set 8:	Scenario A	Scenario B
Symptoms	Moderate	Severe
Duration	3 days	5 days
The onset time of antibiotic	8 hours	2 hours
Intensity of symptom relief after antibiotics	50%	90%
Risk of side effects or antibiotic resistance	Low risk (10%)	Moderate risk（50%）
Time required to obtain antibiotics	4 hours	2 hours
Total cost	400 yuan	250 yuan

①In which situation am I more inclined to use antibiotics for my child?

☐ Scenario A ☐ Scenario B ☐ Neither

②In which situation am I more likely to directly purchase antibiotics for my child from a pharmacy?

☐ Scenario A ☐ Scenario B ☐ Neither

Internal consistency choice set	Scenario A	Scenario B
Symptoms	Moderate	Severe
Duration	5 days	3 days
The onset time of antibiotic	2 hours	4 hours
Intensity of symptom relief after antibiotics	90%	50%
Risk of side effects or antibiotic resistance	No risk (0%)	Moderate risk（50%）
Time required to obtain antibiotics	4 hours	0.5 hours
Total cost	250 yuan	400 yuan

①In which situation am I more inclined to use antibiotics for my child?

☐ Scenario A ☐ Scenario B ☐ Neither

②In which situation am I more likely to directly purchase antibiotics for my child from a pharmacy?

☐ Scenario A ☐ Scenario B ☐ Neither

2. Please rank the following 7 factors that may influence your decision to use antibiotics for your child in order of importance from high to low, and fill in the numbers in parentheses (1 being the most important factor, 2 being the second most important factor, and so on, ranking from 1 to 7).

（　）Symptoms

（　）Duration

（　）The onset time of antibiotic

（　）Intensity of symptom relief after antibiotics

（　）Risk of side effects or antibiotic resistance

（　）Time required to obtain antibiotics

（　）Total cost

Part 1: Survey on Antibiotic Usage Decision-Making by Parents of Children with Colds (Block 2)

1. This part consists of 9 scenarios, each with two situations. This section focuses on how you weigh the decision-making process of using antibiotics when your child has a cold. There are no correct answers for each question. Based on your experience, please assess the characteristics of Scenario A and Scenario B and decide whether to use antibiotics for your child. Please tick only one box for each question.

Choice set 1:	Scenario A	Scenario B
Symptoms	Mild	Moderate
Duration	1 day	5 days
The onset time of antibiotic	4 hours	2 hours
Intensity of symptom relief after antibiotics	90%	50%
Risk of side effects or antibiotic resistance	No risk (0%)	Low risk（10%）
Time required to obtain antibiotics	4 hours	2 hours
Total cost	250 yuan	50 yuan

①In which situation am I more inclined to use antibiotics for my child?

☐ Scenario A　　☐ Scenario B　　☐ Neither

②In which situation am I more likely to directly purchase antibiotics for my child from a pharmacy?

☐ Scenario A　　☐ Scenario B　　☐ Neither

Choice set 2:	Scenario A	Scenario B
Symptoms	Severe	Moderate
Duration	5 days	7 days
The onset time of antibiotic	2 hours	4 hours
Intensity of symptom relief after antibiotics	50%	90%
Risk of side effects or antibiotic resistance	Moderate risk (50%)	No risk（0%）
Time required to obtain antibiotics	4 hours	6 hours
Total cost	150 yuan	50 yuan

①In which situation am I more inclined to use antibiotics for my child?

☐ Scenario A ☐ Scenario B ☐ Neither

②In which situation am I more likely to directly purchase antibiotics for my child from a pharmacy?

☐ Scenario A ☐ Scenario B ☐ Neither

Choice set 3:	Scenario A	Scenario B
Symptoms	Severe	Mild
Duration	3 days	5 days
The onset time of antibiotic	2 hours	8 hours
Intensity of symptom relief after antibiotics	90%	50%
Risk of side effects or antibiotic resistance	No risk (0%)	Moderate risk（50%）
Time required to obtain antibiotics	2 hours	6 hours
Total cost	400 yuan	150 yuan

①In which situation am I more inclined to use antibiotics for my child?

☐ Scenario A ☐ Scenario B ☐ Neither

②In which situation am I more likely to directly purchase antibiotics for my child from a pharmacy?

☐ Scenario A ☐ Scenario B ☐ Neither

Choice set 4:	Scenario A	Scenario B
Symptoms	Mild	Moderate
Duration	3 days	7 days
The onset time of antibiotic	2 hours	4 hours
Intensity of symptom relief after antibiotics	90%	50%
Risk of side effects or antibiotic resistance	Moderate risk (50%)	High risk（90%）
Time required to obtain antibiotics	6 hours	0.5 hours
Total cost	50 yuan	250 yuan

①In which situation am I more inclined to use antibiotics for my child?

☐ Scenario A ☐ Scenario B ☐ Neither

②In which situation am I more likely to directly purchase antibiotics for my child from a pharmacy?

☐ Scenario A ☐ Scenario B ☐ Neither

Choice set 5:	Scenario A	Scenario B
Symptoms	Severe	Moderate
Duration	3 days	1 days
The onset time of antibiotic	8 hours	4 hours
Intensity of symptom relief after antibiotics	90%	50%
Risk of side effects or antibiotic resistance	No risk（0%）	High risk (90%)
Time required to obtain antibiotics	0.5 hours	4 hours
Total cost	250 yuan	400 yuan

①In which situation am I more inclined to use antibiotics for my child?

☐ Scenario A ☐ Scenario B ☐ Neither

②In which situation am I more likely to directly purchase antibiotics for my child from a pharmacy?

☐ Scenario A ☐ Scenario B ☐ Neither

Choice set 6:	Scenario A	Scenario B
Symptoms	Mild	Severe
Duration	3 days	1 day
The onset time of antibiotic	8 hours	2 hours
Intensity of symptom relief after antibiotics	50%	90%
Risk of side effects or antibiotic resistance	High risk（90%）	Low risk (10%)
Time required to obtain antibiotics	2 hours	6 hours
Total cost	50 yuan	400 yuan

①In which situation am I more inclined to use antibiotics for my child?

☐ Scenario A ☐ Scenario B ☐ Neither

②In which situation am I more likely to directly purchase antibiotics for my child from a pharmacy?

☐ Scenario A ☐ Scenario B ☐ Neither

Choice set 7:	Scenario A	Scenario B
Symptoms	Severe	Moderate
Duration	7 days	1 day
The onset time of antibiotic	4 hours	8 hours
Intensity of symptom relief after antibiotics	50%	90%
Risk of side effects or antibiotic resistance	No risk（0%）	Low risk (10%)
Time required to obtain antibiotics	4 hours	0.5 hours
Total cost	150 yuan	250 yuan

①In which situation am I more inclined to use antibiotics for my child?

☐ Scenario A ☐ Scenario B ☐ Neither

②In which situation am I more likely to directly purchase antibiotics for my child from a pharmacy?

☐ Scenario A ☐ Scenario B ☐ Neither

Choice set 8:	Scenario A	Scenario B
Symptoms	Mild	Moderate
Duration	1 day	7 days
The onset time of antibiotic	4 hours	8 hours
Intensity of symptom relief after antibiotics	50%	90%
Risk of side effects or antibiotic resistance	High risk (90%)	No risk（0%）
Time required to obtain antibiotics	2 hours	0.5 hours
Total cost	250 yuan	150 yuan

①In which situation am I more inclined to use antibiotics for my child?

☐ Scenario A ☐ Scenario B ☐ Neither

②In which situation am I more likely to directly purchase antibiotics for my child from a pharmacy?

☐ Scenario A ☐ Scenario B ☐ Neither

Internal consistency choice set	Scenario A	Scenario B
Symptoms	Severe	Moderate
Duration	3 days	1 day
The onset time of antibiotic	8 hours	4 hours
Intensity of symptom relief after antibiotics	90%	50%
Risk of side effects or antibiotic resistance	No risk (0%)	High risk（90%）
Time required to obtain antibiotics	0.5 hours	4 hours
Total cost	250 yuan	400 yuan

①In which situation am I more inclined to use antibiotics for my child?

☐ Scenario A ☐ Scenario B ☐ Neither

②In which situation am I more likely to directly purchase antibiotics for my child from a pharmacy?

☐ Scenario A ☐ Scenario B ☐ Neither

2. Please rank the following 7 factors that may influence your decision to use antibiotics for your child in order of importance from high to low, and fill in the numbers in parentheses (1 being the most important factor, 2 being the second most important factor, and so on, ranking from 1 to 7).

（　）Symptoms

（　）Duration

（　）The onset time of antibiotic

（　）Intensity of symptom relief after antibiotics

（　）Risk of side effects or antibiotic resistance

（　）Time required to obtain antibiotics

（　）Total cost

Part 1: Survey on Antibiotic Usage Decision-Making by Parents of Children with Colds (Block 3)

1. This part consists of 9 scenarios, each with two situations. This section focuses on how you weigh the decision-making process of using antibiotics when your child has a cold. There are no correct answers for each question. Based on your experience, please assess the characteristics of Scenario A and Scenario B and decide whether to use antibiotics for your child. Please tick only one box for each question.

Choice set 1:	Scenario A	Scenario B
Symptoms	Moderate	Severe
Duration	1 day	5 days
The onset time of antibiotic	2 hours	8 hours
Intensity of symptom relief after antibiotics	90%	50%
Risk of side effects or antibiotic resistance	Moderate risk (50%)	Low risk（10%）
Time required to obtain antibiotics	6 hours	4 hours
Total cost	150 yuan	50 yuan

①In which situation am I more inclined to use antibiotics for my child?

☐ Scenario A　　☐ Scenario B　　☐ Neither

②In which situation am I more likely to directly purchase antibiotics for my child from a pharmacy?

☐ Scenario A　　☐ Scenario B　　☐ Neither

Choice set 2:	Scenario A	Scenario B
Symptoms	Severe	Mild
Duration	7 days	5 days
The onset time of antibiotic	8 hours	4 hours
Intensity of symptom relief after antibiotics	50%	90%
Risk of side effects or antibiotic resistance	High risk (90%)	Low risk（10%）
Time required to obtain antibiotics	0.5 hours	4 hours
Total cost	150 yuan	50 yuan

①In which situation am I more inclined to use antibiotics for my child?

☐ Scenario A ☐ Scenario B ☐ Neither

②In which situation am I more likely to directly purchase antibiotics for my child from a pharmacy?

☐ Scenario A ☐ Scenario B ☐ Neither

Choice set 3:	Scenario A	Scenario B
Symptoms	Severe	Mild
Duration	1 day	3 days
The onset time of antibiotic	8 hours	2 hours
Intensity of symptom relief after antibiotics	90%	50%
Risk of side effects or antibiotic resistance	High risk (90%)	Moderate risk（50%）
Time required to obtain antibiotics	6 hours	2 hours
Total cost	50 yuan	400 yuan

①In which situation am I more inclined to use antibiotics for my child?

☐ Scenario A ☐ Scenario B ☐ Neither

②In which situation am I more likely to directly purchase antibiotics for my child from a pharmacy?

☐ Scenario A ☐ Scenario B ☐ Neither

Choice set 4:	Scenario A	Scenario B
Symptoms	Moderate	Severe
Duration	3 days	7 days
The onset time of antibiotic	4 hours	2 hours
Intensity of symptom relief after antibiotics	50%	90%
Risk of side effects or antibiotic resistance	Low risk (10%)	No risk（0%）
Time required to obtain antibiotics	2 hours	4 hours
Total cost	150 yuan	400 yuan

①In which situation am I more inclined to use antibiotics for my child?

☐ Scenario A ☐ Scenario B ☐ Neither

②In which situation am I more likely to directly purchase antibiotics for my child from a pharmacy?

☐ Scenario A ☐ Scenario B ☐ Neither

Choice set 5:	Scenario A	Scenario B
Symptoms	Mild	Severe
Duration	5 days	3 days
The onset time of antibiotic	8 hours	2 hours
Intensity of symptom relief after antibiotics	90%	50%
Risk of side effects or antibiotic resistance	No risk（0%）	High risk (90%)
Time required to obtain antibiotics	2 hours	6 hours
Total cost	400 yuan	250 yuan

①In which situation am I more inclined to use antibiotics for my child?

☐ Scenario A ☐ Scenario B ☐ Neither

②In which situation am I more likely to directly purchase antibiotics for my child from a pharmacy?

☐ Scenario A ☐ Scenario B ☐ Neither

Choice set 6:	Scenario A	Scenario B
Symptoms	Moderate	Mild
Duration	5 days	7 days
The onset time of antibiotic	4 hours	2 hours
Intensity of symptom relief after antibiotics	50%	90%
Risk of side effects or antibiotic resistance	Moderate risk（50%）	High risk（90%）
Time required to obtain antibiotics	0.5 hours	2 hours
Total cost	400 yuan	50 yuan

①In which situation am I more inclined to use antibiotics for my child?

☐ Scenario A ☐ Scenario B ☐ Neither

②In which situation am I more likely to directly purchase antibiotics for my child from a pharmacy?

☐ Scenario A ☐ Scenario B ☐ Neither

Choice set 7:	Scenario A	Scenario B
Symptoms	Moderate	Mild
Duration	7 days	1 day
The onset time of antibiotic	2 hours	8 hours
Intensity of symptom relief after antibiotics	50%	90%
Risk of side effects or antibiotic resistance	Low risk（10%）	High risk (90%)
Time required to obtain antibiotics	6 hours	0.5 hours
Total cost	250 yuan	400 yuan

①In which situation am I more inclined to use antibiotics for my child?

☐ Scenario A ☐ Scenario B ☐ Neither

②In which situation am I more likely to directly purchase antibiotics for my child from a pharmacy?

☐ Scenario A ☐ Scenario B ☐ Neither

Choice set 8:	Scenario A	Scenario B
Symptoms	Mild	Moderate
Duration	7 days	1 day
The onset time of antibiotic	4 hours	8 hours
Intensity of symptom relief after antibiotics	90%	50%
Risk of side effects or antibiotic resistance	Low risk (10%)	No risk（0%）
Time required to obtain antibiotics	0.5 hours	2 hours
Total cost	250 yuan	150 yuan

①In which situation am I more inclined to use antibiotics for my child?

☐ Scenario A ☐ Scenario B ☐ Neither

②In which situation am I more likely to directly purchase antibiotics for my child from a pharmacy?

☐ Scenario A ☐ Scenario B ☐ Neither

Internal consistency choice set	Scenario A	Scenario B
Symptoms	Mild	Severe
Duration	5 days	3 days
The onset time of antibiotic	8 hours	2 hours
Intensity of symptom relief after antibiotics	90%	50%
Risk of side effects or antibiotic resistance	No risk (0%)	High risk（90%）
Time required to obtain antibiotics	2 hours	6 hours
Total cost	400 yuan	250 yuan

①In which situation am I more inclined to use antibiotics for my child?

☐ Scenario A ☐ Scenario B ☐ Neither

②In which situation am I more likely to directly purchase antibiotics for my child from a pharmacy?

☐ Scenario A ☐ Scenario B ☐ Neither

2. Please rank the following 7 factors that may influence your decision to use antibiotics for your child in order of importance from high to low, and fill in the numbers in parentheses (1 being the most important factor, 2 being the second most important factor, and so on, ranking from 1 to 7).

（　）Symptoms

（　）Duration

（　）The onset time of antibiotic

（　）Intensity of symptom relief after antibiotics

（　）Risk of side effects or antibiotic resistance

（　）Time required to obtain antibiotics

（　）Total cost

Part 2: Parents' knowledge of antibiotic use

Knowledge of antibiotic use	Right	Wrong	Don't know
1. Antibiotics are effective in treating most colds.	☐	☐	☐
2. Antibiotics are synonymous with inflammatory drugs.	☐	☐	☐
3. Antibiotics are effective in treating viral colds.	☐	☐	☐
4. Antibiotics are effective in treating bacterial colds.	☐	☐	☐
5. The human body develops resistance to antibiotics.	☐	☐	☐
6. Bacteria develop resistance to antibiotics.	☐	☐	☐
7. Overuse of antibiotic can lead to antibiotic resistance.	☐	☐	☐
8. As long as the usage is short, there will be no antibiotic resistance.	☐	☐	☐

**Self-efficacy**	**Strongly agree**	**Agree**	**Neutral**	**Disagree**	**Strongly disagree**
I think I know enough about the rational use of antibiotics.	☐	☐	☐	☐	☐
I think I'm capable of taking antibiotics to deal with the milder symptoms on my own.	☐	☐	☐	☐	☐
I usually have confidence in self-diagnosis and the treatment of colds on my own.	☐	☐	☐	☐	☐
I usually know when antibiotics are needed.	☐	☐	☐	☐	☐
I usually know if I need antibiotics for a cold before I go to doctor.	☐	☐	☐	☐	☐

**Social influence**	**Never**	**Occasionally**	**Sometimes**	**Often**	**Always**
The doctor prescribed antibiotics for my cold in the past.	☐	☐	☐	☐	☐

Friends and family recommended that I use antibiotics to treat my cold.	☐	☐	☐	☐	☐
The pharmacy recommended that I purchase antibiotics to treat my cold.	☐	☐	☐	☐	☐
The antibiotic use for treating or preventing a cold is a common practice.	☐	☐	☐	☐	☐
**Antibiotic use habit in the past year**	**Never**	**Occasionally**	**Sometimes**	**Often**	**Always**
Have you ever used antibiotics to prevent disease deterioration when the illness was not yet severe?	☐	☐	☐	☐	☐
Have you ever taken antibiotics before seeking medical attention?	☐	☐	☐	☐	☐
Have you ever purchased and self-administered antibiotics from a pharmacy without seeking medical care?	☐	☐	☐	☐	☐
Do you keep antibiotics readily available in your household?	☐	☐	☐	☐	☐
**Availability of antibiotics**	**Never**	**Occasionally**	**Sometimes**	**Often**	**Always**
I can easily purchase antibiotics from a pharmacy without prescription.	☐	☐	☐	☐	☐
I have never been asked to provide a prescription when buying antibiotics.	☐	☐	☐	☐	☐
I can easily obtain antibiotics from my family, friends or from home medicine cabinets.	☐	☐	☐	☐	☐

Part 3 Basic Personal Information

1. Gender	☐Male ☐Female
2. Age	________years old
3. Child's Gender	☐Male ☐Female
4. Child's Age	________years old
5. Educational Level	☐Primary School or below ☐Junior High School ☐Senior High School or Vocational School ☐Junior College ☐Bachelor's Degree ☐Master's Degree ☐ Doctoral Degree
6. Occupation	☐ Farmer ☐ Worker ☐ Student ☐ Healthcare Professional ☐Teacher ☐Government, Enterprise, or Institution Employee ☐Self-employed ☐Retired ☐Unemployed ☐Other_____
7.本人或家人是否有医学背景	☐是 ☐否
8. Annual Family Income Level:_______ ten thousand RMB	☐<2 ☐2-4 ☐4-6 ☐6-8 ☐8-10 ☐10-12 ☐12-14 ☐14-16 ☐16-18 ☐18-20 ☐20-22 ☐22-24 ☐24-26 ☐26-28 ☐28-30 ☐>30
9. Type of Medical Insurance	☐Urban Employee Insurance ☐Urban and Rural Resident Insurance ☐Other______
10. Does the individual or family member have a chronic illness?	☐Yes ☐No

11. Child's Health Status	☐Healthy	☐Good	☐Fair	☐Poor	☐Very Poor

Appendix S3 Discrete Choice Experiment Questionnaire Instructions [In Chinese]

Dear Participant,

Greetings! We are graduate students from Tongji Medical College, Huazhong University of Science and Technology. We are currently conducting a survey on the use of antibiotics in children and would like to understand what factors parents consider when using antibiotics for their children's colds. It will take approximately 15 minutes to complete the questionnaire. There are no right or wrong answers; please fill it out based on your actual situation. A small gift will be provided as a token of our appreciation (the gift can be given in advance). We sincerely thank you for your participation!

...

If you are not familiar with what antibiotics are, common ones include penicillin, amoxicillin, and cephalosporins. If you are interested, you can refer to the instructions at the beginning of the questionnaire. We have also prepared some pictures of commonly used antibiotics (color printed photos), which include those prescribed by doctors for colds or purchased at pharmacies. Now, let me explain to you how to fill it out.

I. When your child has a cold and you consider using antibiotics, you might take the following aspects into account. I will briefly introduce these aspects to you (pointing to each line of the table). For example, when your child has a cold, you might decide whether to give them medicine based on the severity of their symptoms (mild/moderate/severe) and the duration (1 day/3 days/5 days/7 days); if you decide to give them medicine, you might also pay attention to how quickly the medicine takes effect (2 hours/4 hours/8 hours), and how much relief the child feels (50%/90%); at the same time, you might consider the likelihood of side effects after taking the medicine (none/low/medium/high risk); of course, you might also consider the time (0.5 hours/2 hours/4 hours/6 hours) and cost (50 yuan/150 yuan/250 yuan/400 yuan) of obtaining antibiotics from a hospital or pharmacy. We want to understand how ordinary parents think about giving their children antibiotics, so we have assumed different possible scenarios for these aspects to see if everyone would consider giving their children antibiotics under different circumstances.

ID	Attribute	Levels	Definition
1	Symptoms	Mild/Moderate/Severe	Indicates the severity of symptoms
2	Duration	1 day/3 days/5 days/7 days	Indicates the number of days symptoms persist, starting from the appearance of symptoms
3	Time to Effect	2 hours/4 hours/8 hours	Indicates the time it takes for the antibiotic to take effect after being taken
4	Symptom Relief Intensity	50% relief/90% relief	Indicates the degree of symptom relief after taking the antibiotic, the higher the probability, the better the relief effect of the antibiotic on symptoms

5	Risk of Side Effects or Resistance	No risk (0%)/Low risk (10%)/Medium risk (50%)/High risk (90%)	Indicates the possibility of adverse reactions or antibiotic resistance after taking the antibiotic. The higher the probability, the higher the risk of this outcome
6	Time to Obtain Antibiotics	0.5 hours/2 hours/4 hours/6 hours	The time spent to obtain antibiotics
7	Total Cost	50 yuan/150 yuan/250 yuan/400 yuan	The total cost incurred in the process of obtaining antibiotics, including transportation costs, medication costs, or costs incurred during the medical consultation process

II. For example, in the following question, assume your child has a cold and has one or more of the common cold symptoms such as nasal congestion, runny nose, sneezing, sore throat, and fever, and that your child may have an infection and may need to take antibiotics. If you face the following two hypothetical scenarios, based on the comparison of the characteristics of the two scenarios, which scenario would you be more likely to choose to use antibiotics for your child?

**Warm-up Scenario**	**Scenario A**	**Scenario B**
Symptoms	Mild	Moderate
Duration	3 days	1 day
Time to Effect	2 hours	4 hours
Symptom Relief Intensity	50%	90%
Risk of Side Effects or Resistance	Medium risk (50%)	Low risk (10%)
Time to Obtain Antibiotics	2 hours	2 hours
Total Cost	150 yuan	50 yuan

①In scenario__________, I would be more inclined to use antibiotics for my child.

☑ Scenario A ☐ Scenario B ☐Neither

②In scenario__________, I am more likely to go directly to the pharmacy to purchase antibiotics for my child.

☐ Scenario A ☑ Scenario B ☐Neither

In this example, Scenario A means that if the child's cold symptoms are mild, lasting for three days, and taking antibiotics now can take effect within 2 hours, reducing half of the symptoms, and there may be a 50% chance of side effects, and it takes 2 hours and 150 yuan to go to the hospital to get a prescription or buy antibiotics from a pharmacy; Scenario B indicates that the child's cold symptoms are moderate, lasting for one day, and if taking antibiotics, they will take effect within 4 hours, reducing 90% of the symptoms (which can basically cure the child), with a lower risk of side effects or resistance, and it takes 2 hours and 50 yuan to go to the hospital or pharmacy to buy antibiotics.

Suppose a respondent chooses Scenario A for the first question and Scenario B for the second question. This means that among these two hypothetical scenarios, after consideration, he is more likely to use antibiotics for his child in Scenario A and more likely to go directly to the pharmacy to buy antibiotics for his child in Scenario B.

Note:

① There will be 9 hypothetical questions like this below that require you to think and make a choice, and you should tick a box for the plan you prefer. Although each plan seems the same, each line is actually different, so please read carefully and choose.

② We have set a duplicate question in the questionnaire, which is exactly the same as a previous question. Please answer carefully, and you can call me at any time during the filling process.

③ If someone asks about the difference between "wanting to give children antibiotics" and "going to the pharmacy to buy antibiotics," the former means that parents want to give their children antibiotics, whether it's leftover at home, prescribed by a doctor, or bought at a pharmacy, while the latter refers to going directly to the pharmacy to buy medicine for children, and getting a prescription from a doctor is not included.

Appendix S4

Table S4-1 Results of main effects and the interaction effects of demographic characteristics as well as antibiotic cognition in the mixed logit model

Attributes	Coefficient	SE	SD	SE
Symptom (Ref: mild)				
Moderate	1.04***	0.15	-0.23	0.32
Severe	1.40***	0.36	1.99***	0.18
Duration (Ref:1day)				
3 days	1.01***	0.19	-0.48***	0.24
5 days	1.12***	0.22	0.09	0.28
7 days	1.23***	0.21	-0.82***	0.18
Antibiotic effectiveness (Ref: 50%)				
90%	0.37***	0.15	0.83***	0.13
Onset time of antibiotic (Ref: 2 hours)	-0.03	0.21	1.12***	0.19
4 hours	-0.45***	0.20	0.34	0.22
8 hours	-1.31***	0.24	1.18***	0.19
Risk of side effect or resistance (Ref: No risk)				
Low risk	0.25	0.29	0.54	0.32
Moderate risk	0.54***	0.20	0.52	0.28
High risk	-0.08	0.23	0.72***	0.20
Time spent obtaining antibiotics (Ref: 0.5 hours）				
2 hours	0.74***	0.25	-0.47***	0.21
4 hours	0.76***	0.17	-0.10	0.20
6 hours	0.72***	0.22	0.03	0.21
Alternative specific constant (ASC)	-0.30	0.65	2.37***	0.20
Interaction term				
Symptom-Severe*mother	0.71***	0.34	-	-
Symptom-Severe*medical background	0.82***	0.35	-	
Symptom-Severe * Undergraduate education and above	0.64***	0.30	-	-
Symptom-Moderate*income- >120000 yuan	0.34	0.18	-	-
Symptom-Severe * income- >120000 yuan	0.53	0.31	-	-
risk-low*girl	0.45***	0.22	-	-
risk-low * income- >120000 yuan	-0.45***	0.21	-	-
risk-moderate *girl	0.29	0.18	-	-
risk-moderate*Undergraduate education and above	-0.31	0.18	-	-
risk-high* high self-efficacy	0.38	0.25	-	-
duration-5 days* high self-efficacy	-0.51***	0.19	-	-
duration-5 days *high antibiotic availability	0.42***	0.19	-	-
duration-7 days * high self-efficacy	-0.50***	0.21	-	-
Onset time-4 hours* high self-efficacy	0.25	0.17	-	-
Onset time -4 hours* Undergraduate education and above	-0.41***	0.17	-	-
Onset time -8 hours*>35years old	-0.36***	0.16	-	-
Total cost-150 yuan* Undergraduate education and above	-0.32	0.23	-	-
Total cost-150 yuan * low knowledge	0.20	0.20	-	-

level of antibiotic				
Total cost-150 yuan * income- >120000 yuan	-0.66***	0.24	-	-
Total cost-250 yuan * income- >120000 yuan	-0.80***	0.21	-	-
Total cost-400 yuan * income- >120000 yuan	-0.73***	0.24	-	-
Total cost-400 yuan * Undergraduate education and above	-0.19	0.22	-	-
Total cost-400 yuan *high social influence	0.54***	0.23	-	-
Time-2 hours* medical background	-0.60***	0.27	-	-
Time-2 hours * Undergraduate education and above	-0.46***	0.23	-	-
Time-2 hours *low knowledge level of antibiotic	0.33	0.23	-	-
Time-2 hours * high self-efficacy	0.53***	0.22	-	-
Time-4 hours* Undergraduate education and above	-0.52***	0.22	-	-
Time-6 hours * low knowledge level of antibiotic	0.62***	0.20	-	-
